# Integrated Physiological, Transcriptomic, and Metabolomic Analyses Reveal Coordinated Responses of the Invasive Plant *Pistia stratiotes* to Copper Oxide Nanoparticles

**DOI:** 10.3390/plants15213271

**Published:** 2026-10-23

**Authors:** Yuan Zhu, Wen-Ke Ji, Jian-Pan Xin, Pei-Pei Zhong, Chu Zhao, Ying-Hua Jia, Han-Wen Xiao, Ru-Nan Tian

**Affiliations:** 1 College of Landscape Architecture, Nanjing Forestry University, Nanjing 210037, China; zhuy@njfu.edu.cn (Y.Z.); wenkejiii@outlook.com (W.-K.J.); jianpanxin@njfu.edu.cn (J.-P.X.); jkskiki@163.com (P.-P.Z.); jiayinghuajyh@163.com (Y.-H.J.); 2 College of Landscape and Horticulture, Wuhu Vocational Technical University, Wuhu 241002, China; zhaochu@njfu.edu.cn; 3 School of Low Altitude Technology and Geospatial Informatics, Shangqiu Normal University, Shangqiu 476000, China; hwxiao777@163.com

**Keywords:** *Pistia stratiotes*, copper oxide nanoparticles, aquatic ecotoxicology, phytoremediation, photosynthesis, oxidative stress, transcriptomics, metabolomics

## Abstract

To characterize the responses of *Pistia stratiotes* to copper oxide nanoparticles (CuO NPs), plants were exposed to CuO NPs at nominal Cu concentrations of 0 (CK), 0.1 (T_1_), 1.0 (T_2_), 5.0 (T_3_), and 15.0 (T_4_) mg·L^−1^. Cu accumulation and translocation, growth, photosynthetic performance, reactive oxygen species (ROS), antioxidant responses, and molecular profiles were assessed. CuO NP exposure increased root Cu accumulation and reduced Cu translocation to leaves. Relative to CK, the bioconcentration factor (BCF) increased by 301.93% at T_1_ but decreased by 61.65% and 73.69% at T_3_ and T_4_, respectively. At T_1_, leaf chlorophyll and carotenoid contents increased, while photosynthetic performance and most chlorophyll fluorescence parameters remained largely unchanged. Nevertheless, root dry weight, relative growth rate, and stress tolerance index declined. At T_3_ and T_4_, net photosynthetic rate (*P*_n_) decreased by 42.62–60.88% and 52.21–60.86%, respectively, accompanied by increased superoxide (O_2_·^−^) levels. Antioxidant enzyme activities also responded to exposure, while glutathione (GSH) and cysteine (Cys) contents increased at higher concentrations. Transcriptomic and metabolomic analyses of leaves after 14 d were conducted for CK, T_1_, and T_3_. The CK-vs.-T_1_ and CK-vs.-T_3_ comparisons identified 588 and 1088 differentially expressed genes (DEGs) and 166 and 352 differentially accumulated metabolites (DAMs), respectively. Comparison of transcriptomic and metabolomic results suggested associations with pectin-related cell wall processes, carotenoid biosynthesis, and hormone-related metabolism, including changes in the expression of *NCED*1 and other related genes, as well as in ABA-associated metabolites. Overall, the results reveal contrasting physiological responses to low and higher CuO NPs expose concentrations and identify cell wall-, carotenoid-, and hormone-related pathways for further functional investigation.

## 1. Introduction

Aquatic plants have been widely studied for their potential in phytoremediation because they can remove excess nutrients and take up or accumulate potentially toxic elements from contaminated waters [1,2,3,4,5,6]. Their application has been explored in different aquatic treatment systems, highlighting their relevance to water quality improvement. However, the effectiveness of phytoremediation depends not only on contaminant removal but also on the ability of plants to tolerate exposure and maintain physiological function. Changes in growth, photosynthetic performance, mineral nutrient homeostasis, and redox regulation may influence plant responses to pollutants and the overall outcome of phytoremediation. Therefore, understanding the relationship between contaminant accumulation and physiological responses is important for evaluating the suitability of aquatic plants for the treatment of contaminated water.

Copper (Cu) is an essential micronutrient that serves as a cofactor in proteins involved in photosynthetic and respiratory electron transport, ethylene perception, cell-wall metabolism, and antioxidant defense [7,8]. However, excessive Cu exposure can induce oxidative stress, impair photosynthetic function, and disrupt cellular and mineral nutrient homeostasis. Excess Cu can also alter the acquisition and distribution of other mineral nutrients, potentially aggravating physiological dysfunction and growth inhibition [8,9,10,11,12,13,14,15,16,17]. Through these interconnected effects, excess Cu can disrupt multiple physiological processes and ultimately compromise plant growth.

Copper oxide nanoparticles (CuO NPs), which are used in various industrial and technological applications, have attracted attention because of their potential release into aquatic environments and associated ecological risks [18,19,20,21,22,23,24,25]. Once released into aquatic environments, CuO NPs can undergo transformations influenced by factors such as pH, electrolytes, and natural organic matter, potentially altering their dispersion and bioavailability [21,22,23]. Studies of aquatic plants have investigated CuO NPs’ uptake, distribution, and physiological effects under different exposure conditions. In the floating plant *Eichhornia crassipe*s, CuO NPs’ uptake, distribution, transformation, and associated stomatal responses have been examined [26]. Studies involving *Lemna gibba*, *Elodea nuttallii*, and *Lemna mino*r have also investigated the effects of CuO NPs and dissolved Cu on plant growth, photosynthesis, and oxidative stress [27,28,29]. These studies suggest that the contribution of dissolved Cu to CuO NP toxicity can vary with the plant species and exposure conditions. In *L. gibba*, the toxicity of uncoated CuO NPs was attributed mainly to Cu ions released during dissolution [27]. In contrast, in *Landoltia punctata*, CuO NP exposure inhibited growth and reduced chlorophyll content more than could be explained by the measured dissolved Cu concentration alone. These effects were associated with greater Cu accumulation in fronds [30], although this finding does not by itself establish a direct particle-specific mechanism. In addition, CuO NPs have been reported to penetrate root cell walls and be taken up by root cells of *Schoenoplectus tabernaemontani* [31]. At the molecular and metabolic levels, oxylipin-related pathways and glutathione-associated defenses have been implicated in plant stress responses [32,33], while CuO NP exposure has been associated with root cell-wall remodeling in lettuce and distinct physiological and metabolic responses to nanoparticles and dissolved Cu in *Ipomoea aquatica* [34,35]. Collectively, these findings indicate that plant responses to CuO NP exposure may involve both the effects of dissolved Cu released into the exposure medium and processes associated with particle uptake and tissue accumulation. The relative contributions of these processes, however, may vary among plant species and exposure conditions. Despite these advances, further research is needed to clarify how CuO NPs affect different aquatic plant species and how their physiological responses are associated with changes in gene expression and metabolism [36].

*Pistia stratiotes* is a relevant species for phytoremediation research because its free-floating growth form allows its roots to remain in direct contact with the surrounding water, while its biomass can be collected after treatment. Previous studies have evaluated this species in year-round phytofiltration lagoons and field-scale treatment of domestic sewage [37,38]. Its biomass has also been investigated for biofuel production and other forms of utilization [39,40], while broader studies of aquatic macrophytes have examined nutrient removal from waterways [41]. These studies provide application-level evidence for the use of *P. stratiote*s and related aquatic plants in pollutant treatment and biomass utilization. At the physiological and tissue levels, research specifically involving *P. stratiotes* has examined Cd accumulation [42], physiological and metabolic responses to Zn–Cd co-pollution [43], and Mn accumulation in the apoplast under Mn-contaminated conditions [44]. These studies provide complementary evidence of the relevance of *P. stratiotes* to pollutant treatment and metal-stress research, including its potential for metal accumulation, physiological adjustment, and tissue-level metal compartmentation. More recently, Sukul et al. [45] investigated CuO NPs’ uptake and physiological responses in *P. stratiotes* under different nutrient conditions, extending the available evidence on Cu handling and plant responses to CuO NP exposure. However, the transcriptomic and metabolomic changes associated with these responses remain insufficiently characterized, particularly those involving cell-wall- and carotenoid-related pathways and their relationships with tissue Cu distribution and photosynthetic performance.

Despite these advances, how Cu partitioning between roots and leaves relates to the physiological responses of *P. stratiotes* under different CuO NP exposure concentrations and durations remains poorly understood. In addition, the transcriptomic and metabolomic changes associated with these responses, particularly those related to cell-wall processes, carotenoid biosynthesis, and hormone metabolism, have not been fully characterized. To address these gaps, we measured physiological traits and tissue element concentrations across CuO NP treatments and exposure durations and conducted transcriptomic and metabolomic analyses of leaves from CK, T_1_, and T_3_ after 14 d of exposure. Specifically, we aimed to (1) characterize concentration- and time-dependent changes in visible injury, growth, mineral nutrient status, photosynthetic performance, and antioxidant responses; (2) quantify Cu accumulation in roots and leaves and determine the bioconcentration factor (BCF) and translocation factor (TF) across exposure concentrations; and (3) identify candidate genes, metabolites, and pathways associated with the contrasting responses to low and higher CuO NP exposure concentrations. The results indicate that CuO NP exposure elicited a multilevel response involving Cu accumulation and partitioning, photosynthetic function, antioxidant defenses, cell-wall-related metabolism, and carotenoid- and hormone-associated pathways. These responses were concentration dependent, with broader physiological and molecular perturbations at higher concentrations. Because dissolved Cu concentrations were not measured and no soluble-Cu control was included, the observed responses cannot be attributed exclusively to intact CuO NPs.

## 2. Results

### 2.1. Phenotypic Responses in Pistia stratiotes Exposed to CuO NPs

**Figure 1:**
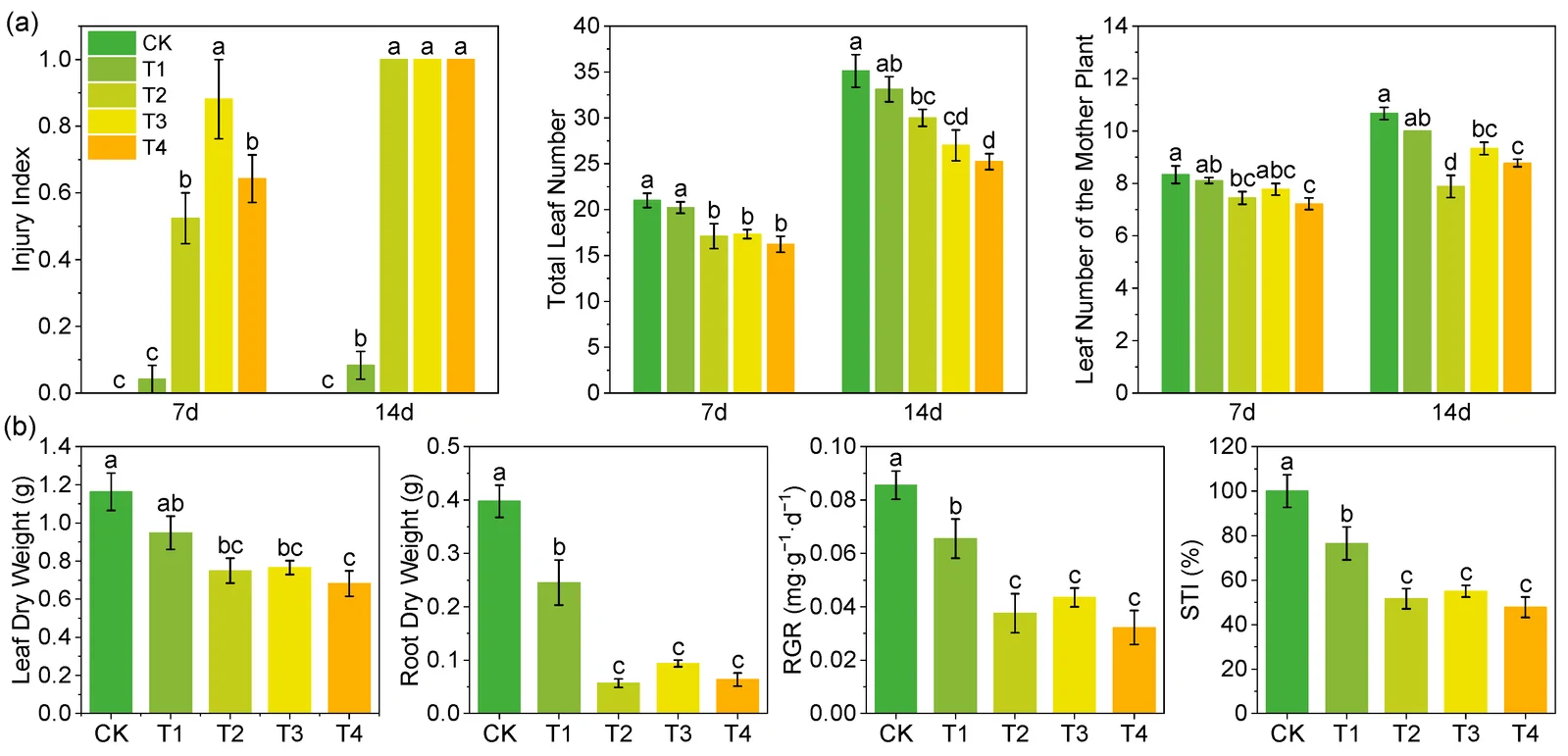
Phenotypic and growth responses of *Pistia stratiotes* to CuO NP exposure. (**a**) Injury index, total leaf number, and leaf number of the mother plants after 7 and 14 d of exposure; (**b**) Leaf dry weight, root dry weight, relative growth rate (RGR), and stress tolerance index (STI) after 14 d of exposure. Data are presented as mean ± SE (*n* = 3). Statistical significance was assessed by one-way ANOVA followed by Duncan’s multiple range test. Different lowercase letters indicate significant differences among treatments (*p* < 0.05). Treatment labels and nominal Cu concentrations are defined as follows: CK, control without CuO NPs; T_1_–T_4_, nominal Cu concentrations of 0.1, 1.0, 5.0, and 15.0 mg Cu·L^−1^, respectively, supplied in the form of CuO NPs. Concentrations are expressed on an elemental-Cu basis. These definitions apply throughout the manuscript and are not repeated in individual figure legends, table footnotes, or Supplementary Materials captions.

The injury index was used to quantify the severity of visible stress symptoms in *Pistia stratiotes* following CuO NP exposure. Total leaf number and the number of leaves on mother plants were also recorded to assess changes in vegetative growth and leaf production. After 7 d of exposure, T_1_ showed no significant differences from CK in the injury index, total leaf number, or the number of leaves on mother plants. In T_2_, the injury index reached 0.54, while total leaf number and the number of leaves on mother plants decreased by 18.52% and 7.43%, respectively. T_3_ showed a similar response in the injury index and total leaf number, whereas the number of leaves on mother plants did not differ significantly from CK. In T_4_, the injury index increased, and both leaf number measures decreased (Figure 1a). After 14 d of exposure, the injury index in T_1_ was 0.08 higher than the CK value, whereas neither total leaf number nor the number of leaves on mother plants differed significantly from CK. In T_2_–T_4_, the injury index reached 1.00, the maximum score on the scale, indicating the highest level of visible injury. Total leaf number and the number of leaves on mother plants decreased by 14.55–28.17% and 12.56–26.05%, respectively (Figure 1a).

The relative growth rate (RGR) and stress tolerance index (STI) were calculated from biomass data to further assess growth responses to CuO NP exposure. RGR reflects the relative rate of biomass increase over time, whereas STI expresses the dry weight of each plant component in a treatment as a percentage of that in the corresponding control. After 14 d of exposure, root dry weight, RGR, and STI were significantly lower in all CuO NP treatments (Figure 1b). In T_1_, root dry weight, RGR, and STI decreased by 37.50%, 22.22%, and 23.56%, respectively, whereas leaf dry weight did not differ significantly from CK. In T_2_–T_4_, leaf and root dry weights decreased by 33.63–41.38% and 60.00–85.00%, respectively, while RGR and STI decreased by 55.56–66.67% and 44.96–52.26%, respectively.

### 2.2. Cu Accumulation and Translocation in P. stratiotes Exposed to CuO NPs

As shown in Table 1, after 14 d of exposure, the total Cu concentration in leaves did not differ significantly from that in CK in T_1_ but increased significantly by 210.05–409.33% in T_2_–T_4_. Root Cu concentrations were 106.17–414.76 times those in CK across the CuO NP treatments, indicating that Cu accumulated predominantly in the roots. The translocation factor (TF), which reflects Cu transfer from roots to leaves, decreased significantly by 76.92–86.15% in T_1_–T_4_. Compared with CK, the bioconcentration factor (BCF), which reflects Cu accumulation relative to its concentration in the exposure medium, increased by 301.93% in T_1_, showed no significant change in T_2_, and decreased by 61.65% and 73.69% in T_3_ and T_4_, respectively.

### 2.3. Changes in Reactive Oxygen Species, Non-Enzymatic Antioxidant Contents, and Antioxidant Enzyme Activities in P. stratiotes Exposed to CuO NPs

Soluble sugar content was measured to assess changes in carbohydrate status, while hydrogen peroxide (H_2_O_2_), superoxide anion (O_2_·^−^), and malondialdehyde (MDA) were measured to characterize ROS accumulation and lipid peroxidation. Antioxidant enzyme activities and non-enzymatic antioxidant contents were also determined to assess the responses of the antioxidant defense system to CuO NP exposure.

**Figure 2:**
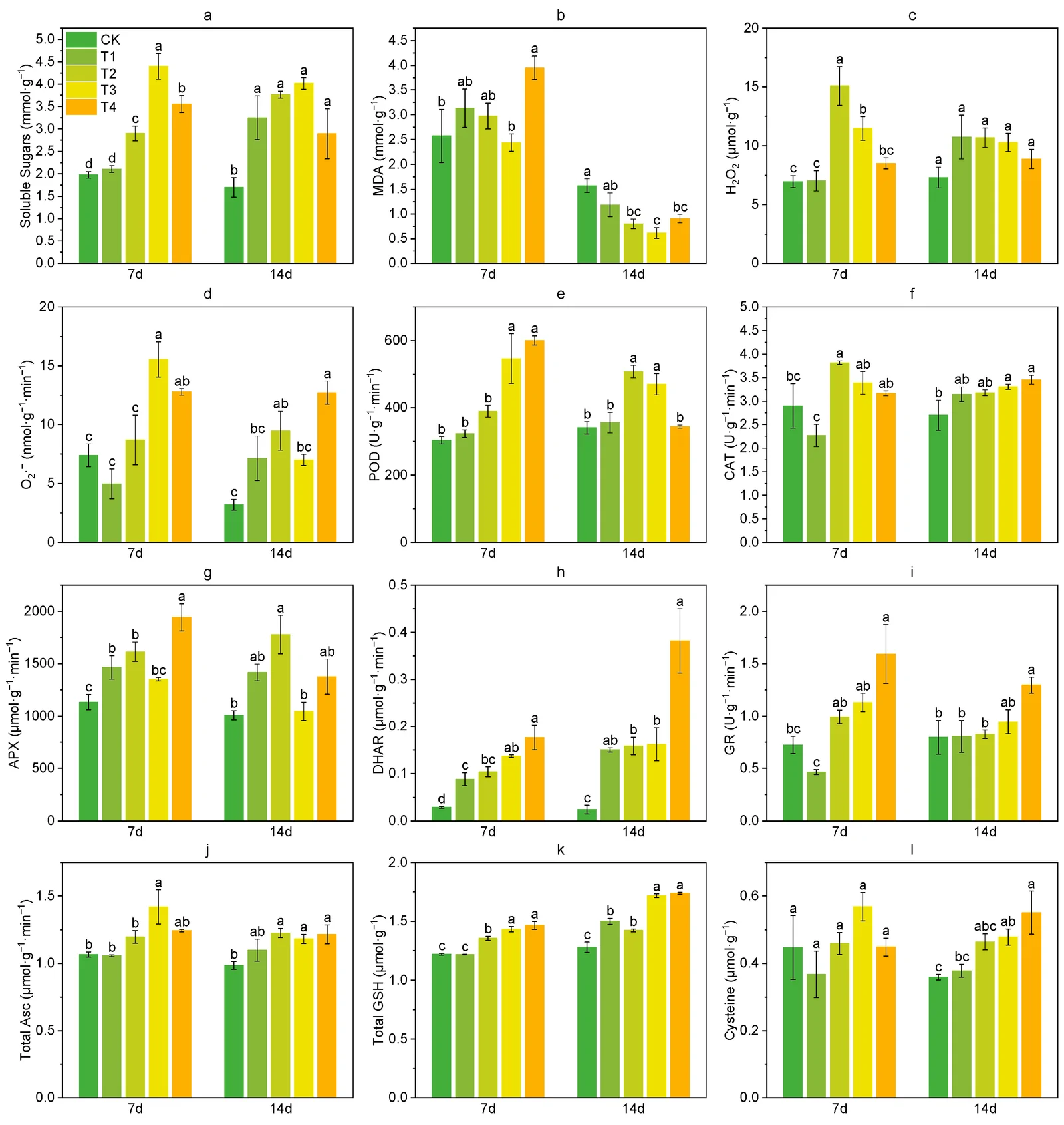
Changes in the soluble sugar content, reactive oxygen species (ROS) levels, lipid peroxidation, and antioxidant responses in *P. stratiotes* after 7 and 14 d of exposure to different concentrations of CuO NPs. (**a**) Soluble sugar content; (**b**) malondialdehyde content (MDA); (**c**) hydrogen peroxide content (H_2_O_2_); (**d**) superoxide anion content (O_2_·^−^); (**e**) peroxidase activity (POD); (**f**) catalase activity (CAT); (**g**) ascorbate peroxidase activity (APX); (**h**) dehydroascorbate reductase activity (DHAR); (**i**) glutathione reductase activity (GR); (**j**) total ascorbate content (total Asc); (**k**) total glutathione content (total GSH); and (**l**) cysteine content. Data are presented as means ± SE (*n* = 3). Different lowercase letters indicate significant differences among treatments at *p* < 0.05, as determined by one-way ANOVA followed by Duncan’s multiple range test.

After 7 d, the soluble sugar content increased by 46.46–122.22% in T_2_–T_4_, while the MDA content increased by 53.70% in T_4_. The H_2_O_2_ content increased by 116.36% and 64.71% in T_2_ and T_3_, respectively, whereas the O_2_·^−^ content increased by 110.70% in T_3_ and 73.30% in T_4_. After 14 d, the soluble sugar content was 70.00–136.47% higher than in CK across all treatments, whereas the MDA content was 42.04–60.51% lower in T_2_–T_4_. At this time point, significant increases in the ROS content were observed only for O_2_·^−^, which increased by 234.63% in T_2_ and 349.47% in T_4_ (Figure 2a–d).

Peroxidase (POD) and catalase (CAT) activities were measured to assess enzymatic H_2_O_2_ scavenging, while ascorbate peroxidase (APX), dehydroascorbate reductase (DHAR), and glutathione reductase (GR) activities were measured to characterize responses of enzymes involved in the ascorbate–glutathione cycle, which contributes to H_2_O_2_ detoxification and the recycling of ascorbate and glutathione. After 7 d, POD activity increased by 80.33% in T_3_ and 98.17% in T_4_, while CAT activity increased by 31.72% in T_2_. APX activity increased by 47.28%, 29.17%, and 71.35% in T_1_, T_2_, and T_4_, respectively, while GR activity increased by 120.83% in T_4_. DHAR activity reached 3.08–6.17 times the CK level across CuO NP treatments. After 14 d, POD activity increased by 49.37% in T_2_ and 38.32% in T_3_, while CAT activity increased by 22.59% in T_3_ and 28.15% in T_4_. APX activity increased by 76.39% in T_2_, and GR activity increased by 62.50% in T_4_. DHAR activity reached 6.30–13.60 times the CK level across treatments (Figure 2e–i).

The total ascorbate (Asc), total glutathione (GSH), and cysteine (Cys) contents were measured to assess changes in non-enzymatic components involved in cellular redox regulation. After 7 d, the total Asc content increased by 32.71% in T_3_, while the total GSH content increased by 11.48–19.67% in T_2_–T_4_. After 14 d, the total Asc content increased by 20.41–25.51% in T_2_–T_4_. In T_3_ and T_4_, the total GSH and Cys contents increased by 27.41–28.89% and 33.33–52.78%, respectively (Figure 2j–l).

### 2.4. Changes in Photosynthetic Pigments and Gas-Exchange Parameters in P. stratiotes Exposed to CuO NPs

Photosynthetic pigment contents (chlorophyll a, Chl a; chlorophyll b, Chl b; and carotenoids, Car) and the Chl/Car ratio were measured to assess changes in pigment abundance and relative composition. The net photosynthetic rate (*P*_n_), intercellular CO_2_ concentration (*C*_i_), and instantaneous carboxylation efficiency (CE) were measured to evaluate photosynthetic performance and determine whether it changed alongside pigment responses.

**Figure 3:**
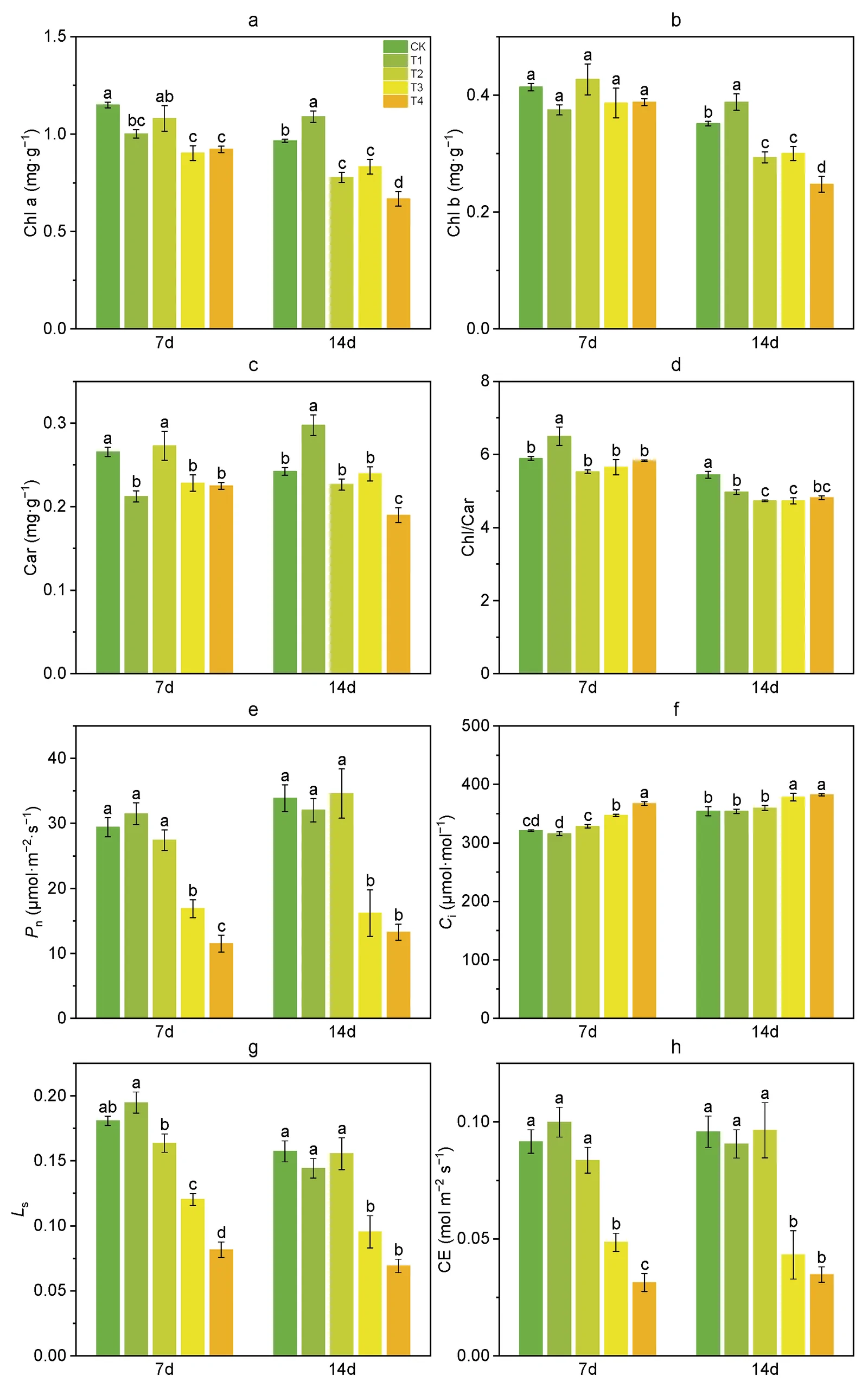
Changes in photosynthetic pigment contents and leaf gas-exchange parameters in *P. stratiotes* after 7 and 14 d of exposure to different concentrations of CuO NPs. (**a**) Chlorophyll a (Chl a) content; (**b**) chlorophyll b (Chl b) content; (**c**) carotenoid (Car) content; (**d**) chlorophyll-to-carotenoid ratio (Chl/Car); (**e**) net photosynthetic rate (*P*_n_); (**f**) intercellular CO_2_ concentration (*C*_i_); (**g**) stomatal limitation (*L*_s_); and (**h**) instantaneous carboxylation efficiency (CE). Data are presented as means ± SE (*n* = 3). Different lowercase letters indicate significant differences among treatments within each exposure duration (*p* < 0.05), as determined by one-way ANOVA followed by Duncan’s multiple range test.

After 7 d, the Chl a content decreased by 12.88% in T_1_ and by 19.76–21.50% in T_3_–T_4_, while the Car content followed a similar trend, and the Chl b content did not differ significantly from CK. The Chl/Car ratio increased by 10.34% in T_1_ (Figure 3a–d). After 14 d, the Chl a and Chl b contents increased by 12.73% and 10.54%, respectively, in T_1_ but decreased by 12.88–21.50% and 14.53–29.63%, respectively, in T_2_–T_4_. The Car content decreased only in T_4_, whereas the Chl/Car ratio decreased by 8.65–13.15% in T_1_–T_4_ relative to CK (Figure 3a–d).

*P*_n_ did not differ significantly from CK in T_1_ or T_2_ at either sampling time but decreased by 42.62–60.88% in T_3_ and 52.21–60.86% in T_4_ (Figure 3e). Stomatal limitation (*L*_s_) showed a similar response pattern across treatments (Figure 3g). After 7 d, *C*_i_ increased by 8.20% in T_3_ and 14.43% in T_4_. CE did not differ significantly from CK in T_1_ or T_2_ but decreased by 46.74% in T_3_ and 66.30% in T_4_. This pattern persisted at 14 d, with lower *P*_n_ and CE and higher *C*_i_ in T_3_ and T_4_ than in CK (Figure 3f,h). Although *P*_n_ and CE did not differ significantly from CK under T_1_, this did not translate into an overall growth benefit, as the root dry weight, RGR, and STI were reduced (Figure 1b).

### 2.5. Changes in Chlorophyll a Fluorescence Parameters in P. stratiotes Exposed to CuO NPs

Chlorophyll a fluorescence was analyzed using the OJIP transient method to characterize PSII photochemistry, energy dissipation, and electron transport, complementing the measurements of photosynthetic pigments, gas-exchange parameters, and ROS and antioxidant responses.

**Figure 4:**
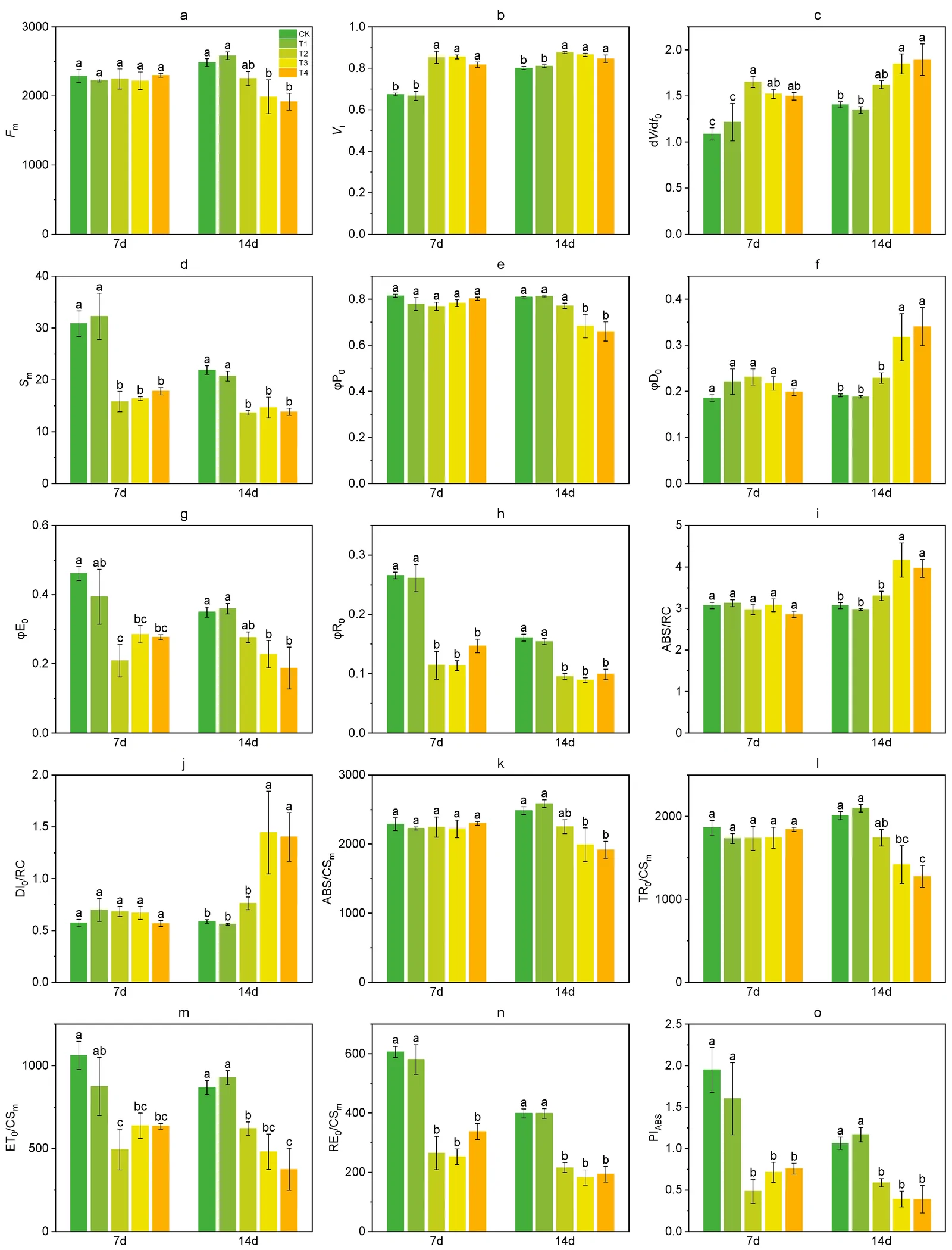
Changes in chlorophyll a fluorescence parameters in *P. stratiotes* after 7 and 14 d of exposure to different concentrations of CuO NPs. (**a**) Maximum fluorescence (*F*_m_); (**b**) I-step (*V*_i_); (**c**) initial slope of the OJIP curve (d*V*/d*t*_0_); (**d**) normalized area above the OJIP transient (*S*_m_); (**e**) maximum photochemical quantum yield (φP_0_); (**f**) quantum yield of non-photochemical dissipation (φD_0_); (**g**) quantum yield for electron transport (φE_0_); (**h**) quantum yield for reduction of terminal acceptors on the PSI acceptor side (φR_0_); (**i**) absorbed light energy flux per reaction center (ABS/RC); (**j**) dissipated light energy flux per reaction center (DI_0_/RC); (**k**) absorbed light energy flux per unit cross-sectional area (ABS/CS_m_); (**l**) trapped light energy flux per unit cross-sectional area (TR_0_/CS_m_); (**m**) electron transport flux per unit cross-sectional area (ET_0_/CS_m_); (**n**) electron flux per unit cross-sectional area for the reduction of terminal electron acceptors on the PSI acceptor side (RE_0_/CS_m_); and (**o**) performance index (PI_ABS_). Data are presented as means ± SE (*n* = 3). Different lowercase letters indicate significant differences among treatments within each exposure duration (*p* < 0.05), as determined by one-way ANOVA followed by Duncan’s multiple range test.

After 7 d of CuO NP exposure, maximum fluorescence (*F*_m_), maximum photochemical quantum yield (φP_0_), quantum yield of non-photochemical dissipation (φD_0_), absorbed light energy flux per reaction center (ABS/RC), dissipated light energy flux per reaction center (DI_0_/RC), absorbed light energy flux per unit cross-sectional area (ABS/CS_m_), and trapped light energy flux per unit cross-sectional area (TR_o_/CS_m_) did not differ significantly from CK (Figure 4a,e,f,i–l). In T_2_–T_4_, relative variable fluorescence at the I-step (*V*_i_) and the initial slope of the OJIP curve (d*V*/d*t*_0_) increased by 16.85–21.22% and 37.59–51.75%, respectively (Figure 4b,c). In contrast, the energy capacity for reduction of the normalized area above the OJIP transient (*S*_m_), quantum yield for electron transport (φE_o_), and quantum yield for reduction of the terminal acceptors of PSI (φR_o_) decreased by 42.25–48.70%, 38.18–54.66%, and 44.74–57.52%, respectively. Electron transport flux per unit cross-sectional area (ET_o_/CS_m_), electron flux per unit cross-sectional area for the reduction of terminal electron acceptors on the PSI acceptor side (RE_o_/CS_m_), and the performance index (PI_ABS_) also decreased by 39.88–53.33%, 44.36–58.31%, and 61.01–75.12%, respectively (Figure 4d,g,h,m–o). Overall, these changes indicate altered OJIP kinetics and reduced electron transport, particularly toward the terminal electron acceptors on the PSI acceptor side, despite the lack of significant changes in several other fluorescence parameters.

After 14 d of exposure, none of the measured fluorescence parameters differed significantly from CK in T_1_. In T_2_–T_4_, *V*_i_ increased, whereas *S*_m_ and φR_0_ decreased; ET_0_/CS_m_, RE_o_/CS_m_, and PI_ABS_ also decreased. More extensive changes occurred in T_3_ and T_4_. In T_3_, *F*_m_, *S*_m_, φP_0_, φE_0_, φR_0_, ABS/CS_m_, TR_0_/CS_m_, ET_0_/CS_m_, RE_0_/CS_m_, and PI_ABS_ decreased by 19.95%, 33.09%, 15.57%, 34.96%, 44.10%, 19.95%, 29.34%, 44.59%, 54.19%, and 63.06%, respectively. In contrast, *V*_i_, d*V*/d*t*_0_, φD_0_, ABS/RC, and DI_0_/RC increased by 7.99%, 31.62%, 39.75%, 35.77%, and 146.00%, respectively. T4 showed the same directional changes (Figure 4a–o). The decreases in φP_0_, φE, and φR_0_ together with the lower ET_0_/CS_m_ and RE_0_/CS_m_ values indicate impaired PSII photochemistry and reduced electron transport. The marked decline in PI_ABS_ further reflects reduced overall PSII performance. Meanwhile, increases in φD_0_ and DI_0_/RC indicate greater energy dissipation, while the increase in ABS/RC suggests an increase in absorbed energy flux per active reaction center. These responses point to altered energy partitioning and impaired photosynthetic electron transport under higher CuO NP exposure.

### 2.6. Metabolomic Analysis

#### 2.6.1. Effects of CuO NPs Stress on Leaf Metabolism in *P. stratiotes*

Untargeted metabolomics was used to identify metabolite-level changes associated with CuO NP exposure and to compare responses under lower and higher exposure levels.

**Figure 5:**
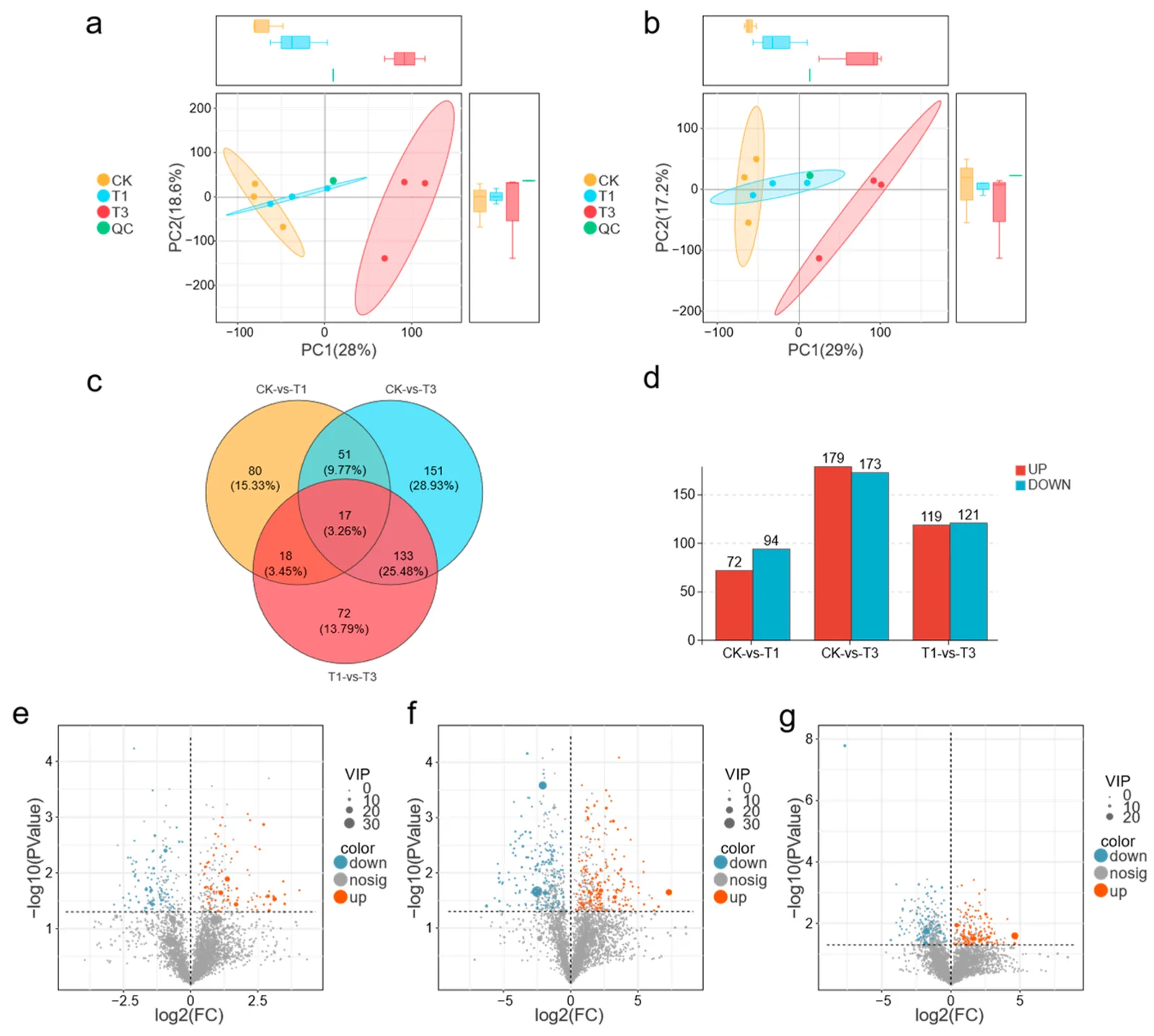
Metabolomic quality control and differential metabolite analysis after 14 d of CuO NP exposure. (**a**,**b**) PCA score plots of metabolomic data obtained in positive-ion (POS) and negative-ion (NEG) modes, respectively; (**c**) Venn diagram showing the overlap of differentially accumulated metabolites (DAMs) among the three pairwise comparisons; (**d**) numbers of DAMs with increased and decreased abundance in each comparison; (**e**–**g**) volcano plots showing DAMs in the CK-vs.-T_1_, CK-vs.-T_3_, and T_1_-vs.-T_3_ comparisons, respectively.

As shown in Figure 5a,b, the QC samples clustered closely in the PCA plots for both POS and NEG modes, indicating good analytical reproducibility. This was further supported by the low median RSDs of peak intensities, which were 2.78% in POS mode and 3.04% in NEG mode. In addition, 95.09% of the features in POS mode and 94.82% in NEG mode had RSDs ≤ 15%. The biological samples generally clustered by treatment group, with separation between the control and CuO NP-treated groups. A total of 2468 and 1719 metabolites were detected in positive-ion (POS) and negative-ion (NEG) modes, respectively. Using a *p* value < 0.05 and a variable importance in projection (VIP) score ≥ 1 as the screening criteria, 522 unique differentially accumulated metabolites (DAMs) were identified across the three pairwise comparisons.

As shown in Figure 5c,d, 166 DAMs were identified in the CK-vs.-T_1_ comparison, including 72 with increased abundance and 94 with decreased abundance. In the CK-vs.-T_3_ comparison, 352 DAMs were identified, with 179 showing increased abundance and 173 showing decreased abundance. The T_1_-vs.-T_3_ comparison yielded 240 DAMs, including 119 with increased abundance and 121 with decreased abundance. Seventeen DAMs were shared among all three comparisons.

**Figure 6:**
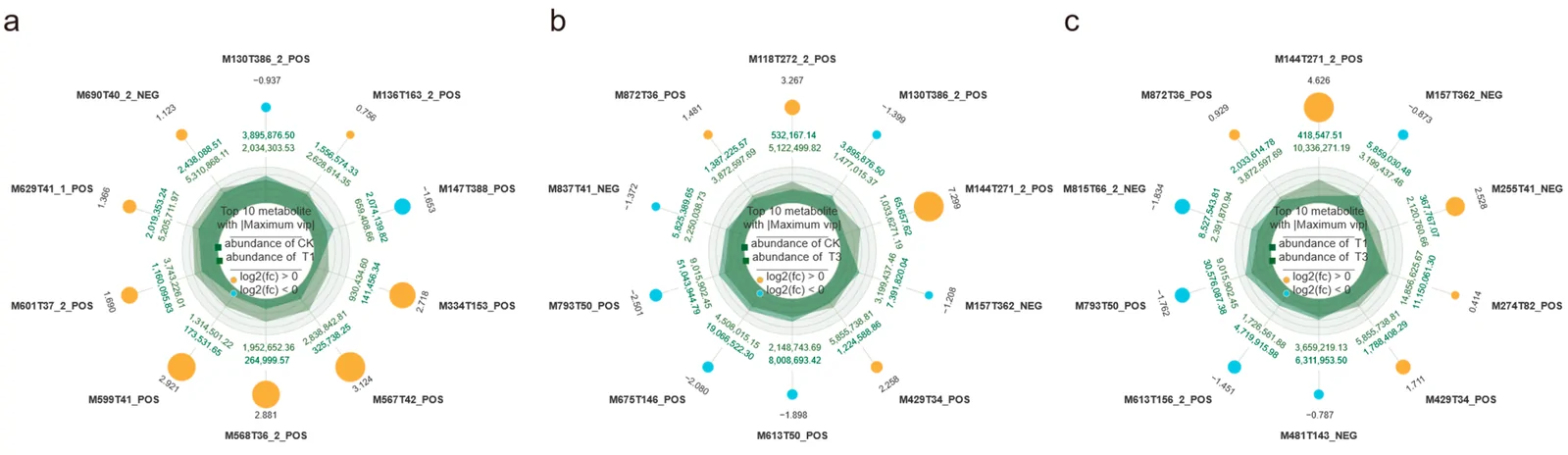
Radar plots of the top 10 DAMs in the (**a**) CK-vs.-T_1_, (**b**) CK-vs.-T_3_, and (**c**) T_1_-vs.-T_3_ comparison groups after 14 d of CuO NP exposure. Values on the outer ring represent log_2_ fold change [log_2_(FC)]. Circle size reflects the magnitude of log_2_(FC), while circle color indicates the direction of change (green, downregulated; blue, upregulated). The two values inside each circle, from top to bottom, represent the mean abundance of the metabolite in the left- and right-hand groups of the corresponding comparison, respectively. The radar polygons represent metabolite abundance profiles across groups; a more pointed extension toward a group indicates a higher abundance of the corresponding metabolite in that group.

For each comparison, the top 10 DAMs ranked by VIP score are presented in Figure 6 and listed in Table S1. In the CK-vs.-T_1_ comparison, 8 of the top 10 DAMs showed increased abundance, including diatoxanthin (M567T42_POS), lutein (M568T36_2_POS), and adenine (M136T163_2_POS). Whereas goitrin (M130T386_2_POS) and DL-glutamine (M147T388_POS) showed decreased abundance.

In the CK-vs.-T_3_ comparison, 4 of the top 10 DAMs showed increased abundance, including stachydrine (M144T271_2_POS), betaine (M118T272_2_POS), and pheophytin a (M872T36_POS); the remaining 6 showed decreased abundance, including nonanoic acid (M157T362_NEG), 2-docosahexaenoyl-1-stearoyl-sn-glycero-3-phosphoethanolamine (SDPE, M793T50_POS), and di-γ-linolenin (6c, 9c, 12c) (M613T50_POS).

In the T_1_-vs.-T_3_ comparison, 5 of the top 10 DAMs showed increased abundance, including stachydrine (M144T271_2_POS), palmitic acid (M255T41_NEG), and pheophytin a (M872T36_POS); the remaining 5 showed decreased abundance, including ginsenoside F3 (M815T66_2_NEG), 2-docosahexaenoyl-1-stearoyl-sn-glycero-3-phosphoethanolamine (SDPE, M793T50_POS), dilinolenin (9c, 12c, 15c) (M613T156_2_POS), nonanoic acid (M157T362_NEG), and silybin (M481T143_NEG).

#### 2.6.2. KEGG Pathway Enrichment Analysis of DAMs

**Figure 7:**
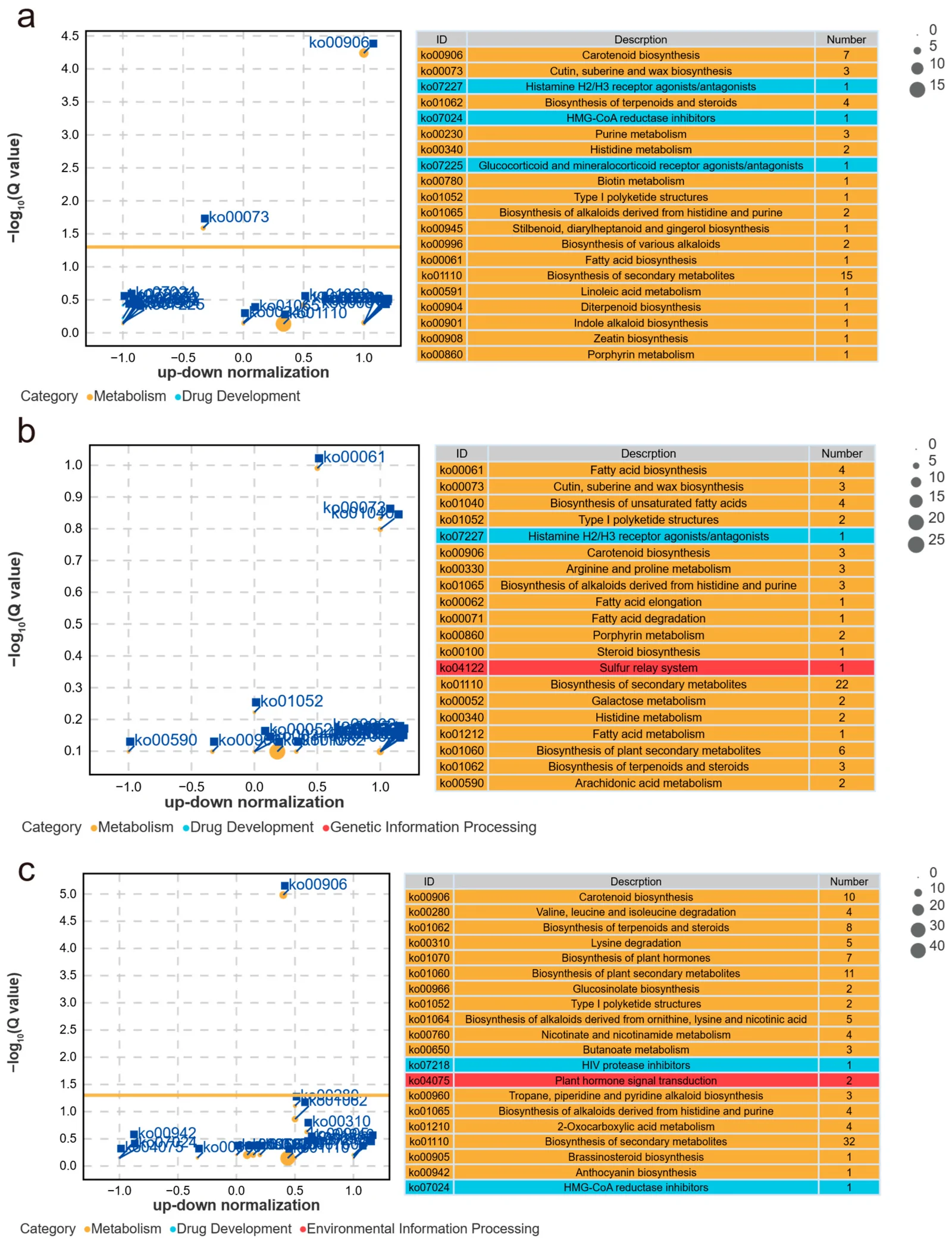
Top 20 KEGG pathways for DAMs in (**a**) CK-vs.-T_1_, (**b**) CK-vs.-T_3_, and (**c**) T_1_-vs.-T_3_ comparison groups after 14 d of CuO NP exposure. Bubble size indicates the number of DAMs enriched in each pathway. The y-axis represents −log_10_(Q value), and the *x*-axis represents the difference between upregulated and downregulated DEM counts divided by the total number of DEGs. The yellow line marks the significance threshold (Q = 0.05; −log_10_(Q) ≈ 1.30); pathways above the line are significantly enriched.

KEGG enrichment analysis revealed that the DAMs were associated with diverse metabolic pathways following CuO NP exposure. Figure 7 shows the top 20 KEGG pathways enriched among the DAMs in each comparison, ranked by *Q* value. In the CK-vs.-T_1_ comparison, the pathways mainly enriched with DAMs included carotenoid biosynthesis (ko00906), cutin, suberin, and wax biosynthesis (ko00073), biosynthesis of terpenoids and steroids (ko01062), purine metabolism (ko00230), and histidine metabolism (ko00340) (Figure 7a).

In the CK-vs.-T_3_ comparison, the main pathways enriched with DAMs included carotenoid biosynthesis (ko00906), biosynthesis of terpenoids and steroids (ko01062), plant hormone biosynthesis (ko01070), lysine degradation (ko00310), 2-oxocarboxylic acid metabolism (ko01210), valine, leucine, and isoleucine degradation (ko00280), and glucosinolate biosynthesis (ko00966) (Figure 7b).

In the T_1_-vs.-T_3_ comparison, the main enriched pathways included fatty acid biosynthesis (ko00061), cutin, suberin and wax biosynthesis (ko00073), biosynthesis of unsaturated fatty acids (ko01040), type I polyketide structures (ko01052), porphyrin metabolism (ko00860), and sulfur relay system (ko04122), among which DAMs in cutin, suberin, and wax biosynthesis, biosynthesis of unsaturated fatty acids, porphyrin metabolism, and sulfur relay system mainly showed increased abundance (Figure 7c).

### 2.7. Transcriptomic Analysis

#### 2.7.1. Transcriptome Sequencing and DEGs Identification

Transcriptome sequencing was used to identify gene-expression changes associated with the physiological and metabolic responses. As shown in Table S2, Q20 ≥ 97.74%, Q30 ≥ 93.37%, and the GC content ranged from 49.78% to 50.83%, indicating the reliability of the transcriptome sequencing results (Table S3). Correlation heatmap analysis of gene expression (Figure S1c–e) revealed that samples from each treatment group formed an independent cluster, and the Pearson correlation coefficients among samples within each group were ≥0.73. This indicated good biological replication, and the obtained sequence data were suitable for subsequent analyses.

**Figure 8:**
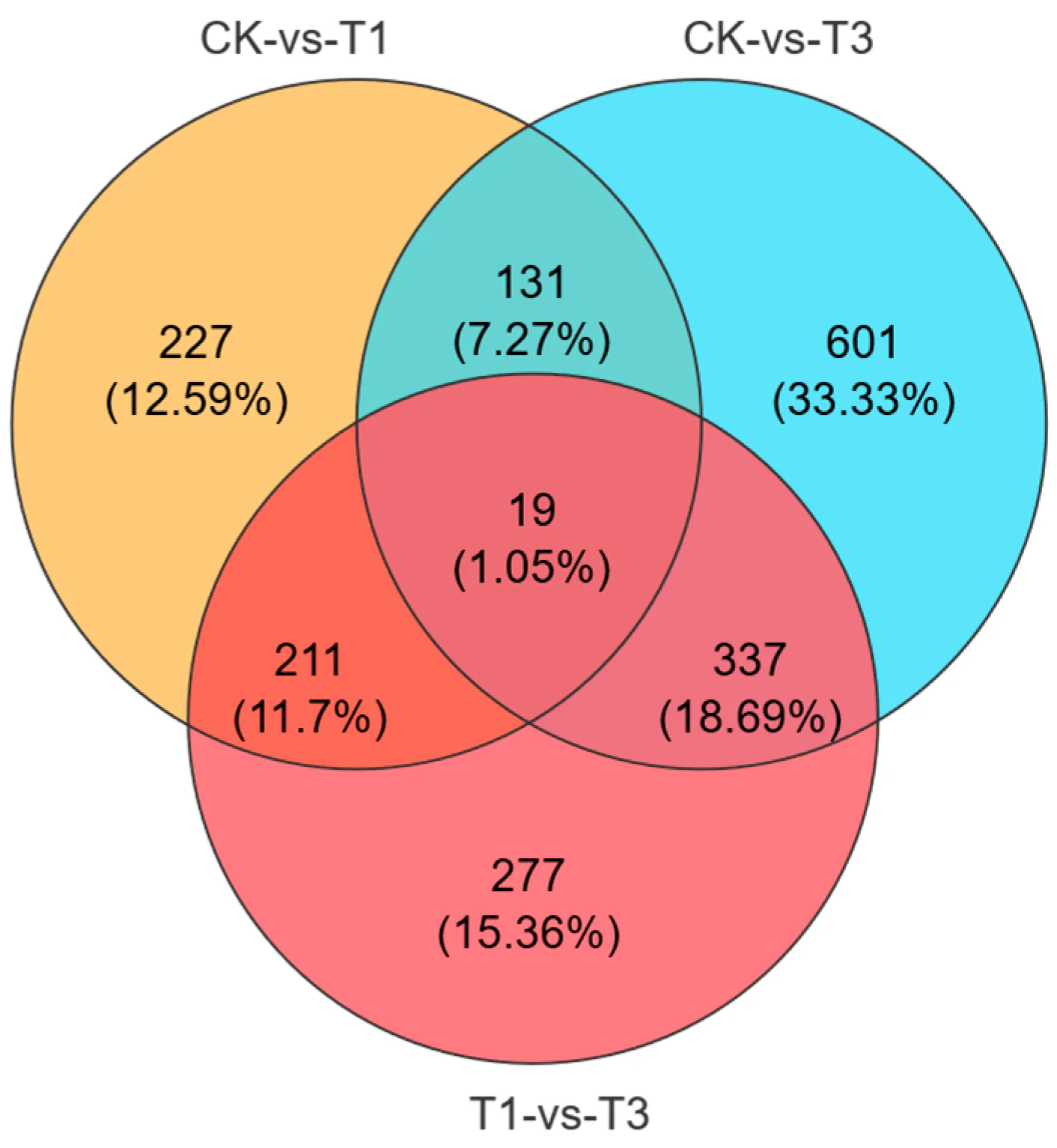
Venn diagram of DEG distribution across the CK-vs.-T_1_, CK-vs.-T_3_, and T_1_-vs.-T_3_ comparison groups after 14 d of CuO NP exposure. The numbers in each region indicate the numbers of DEGs specific to or shared among the respective comparisons.

RNA-seq analysis of plants treated with different concentrations of CuO NPs for 14 d identified a total of 1803 differentially expressed genes (DEGs) in the leaves (Figure 8, Figure S1a). In the CK-vs.-T_1_ comparison, there were 588 DEGs (79 upregulated and 509 downregulated); in the CK-vs.-T_3_ comparison, there were 1088 DEGs (542 upregulated and 546 downregulated); and in the T_1_-vs.-T_3_ comparison, there were 844 DEGs (728 upregulated and 116 downregulated), with 58 DEGs shared among the three comparisons. Notably, DEGs in T_1_-vs.-T_3_ contained substantially more upregulated than downregulated DEGs, with 728 and 116 genes, respectively.

#### 2.7.2. GO and KEGG Pathway Analysis of DEGs

**Figure 9:**
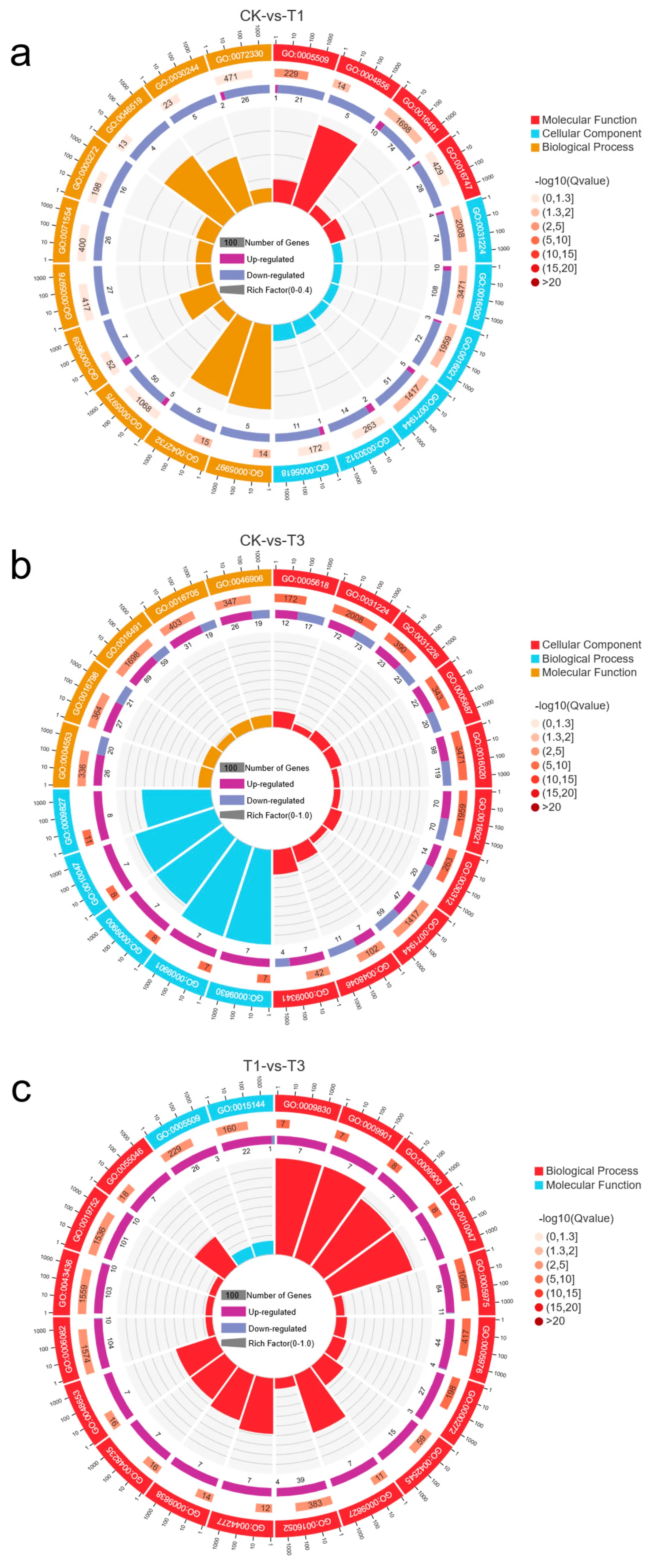

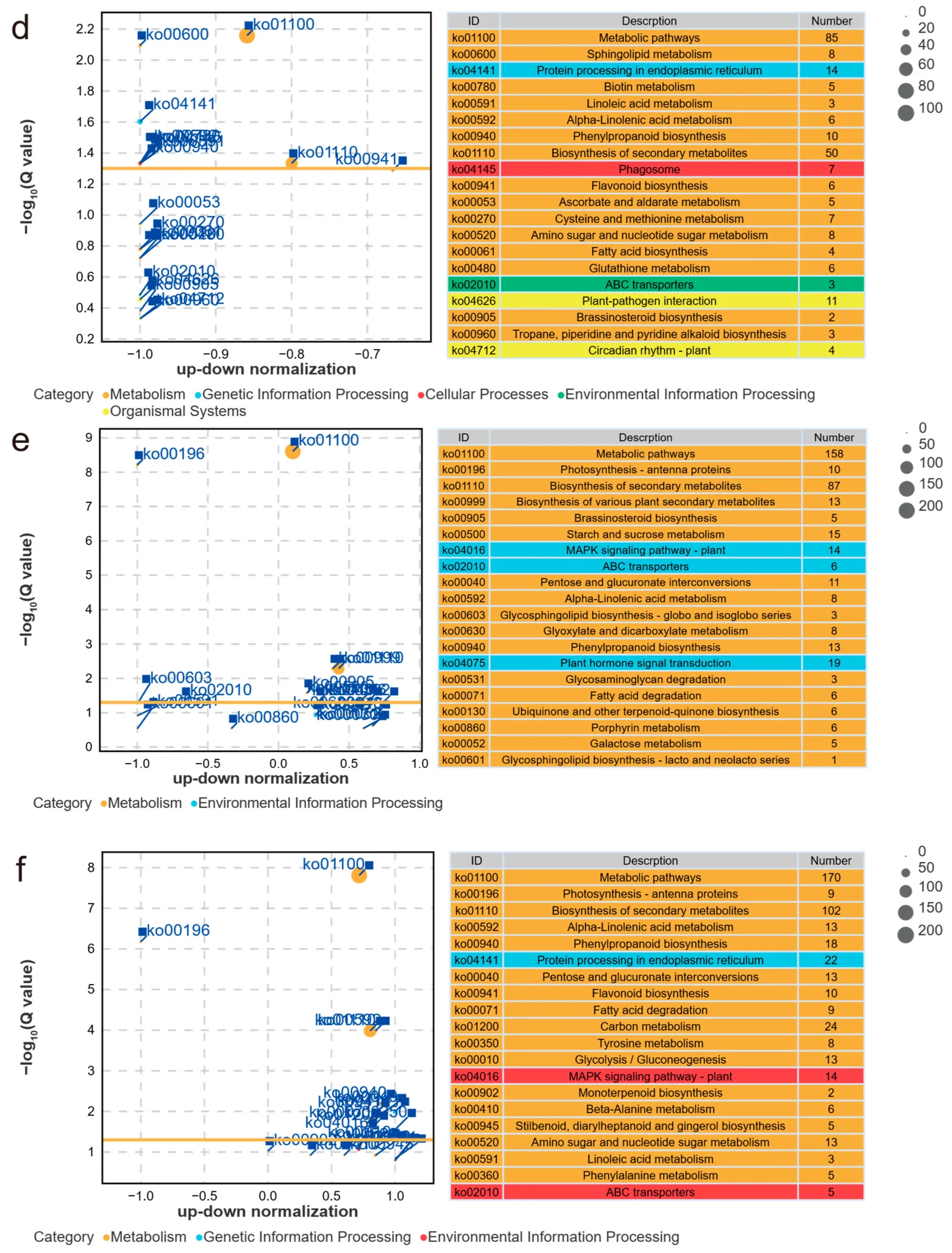
GO enrichment circle plots (**a**–**c**) and KEGG enrichment bubble plots (**d**–**f**). In the GO plots, the outer scale indicates the number of DEGs associated with each GO term. The second ring shows the number of background genes annotated to each term. The background gene set comprises all genes in the reference genome with GO annotations. The third ring shows enriched DEGs, with colors distinguishing upregulated and downregulated genes. The fourth ring represents the Rich factor, calculated as the number of DEGs annotated to a GO term divided by the number of background genes annotated to the same term. In the KEGG plots, bubble size indicates the number of DEGs enriched in each pathway. The *y*-axis represents −log_10_(Q value), and the *x*-axis represents the difference between upregulated and downregulated DEG counts divided by the total number of DEGs. The yellow line marks the significance threshold (Q = 0.05; −log_10_ (Q) ≈ 1.30); pathways above this line are significantly enriched. The numbers of DEGs used for enrichment analysis were 588 for CK-vs.-T_1_, 1088 for CK-vs.-T_3_, and 844 for T_1_-vs.-T_3_.

**Figure 10:**
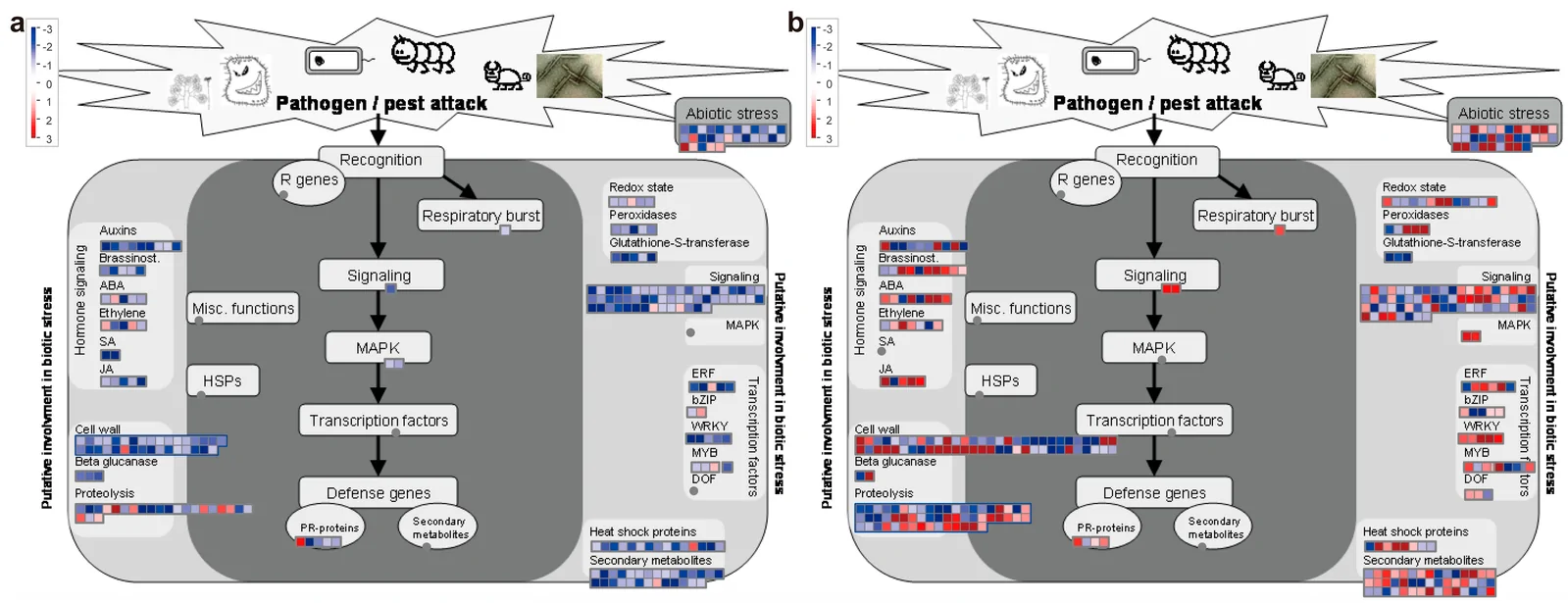
MapMan “biotic stress” overview of DEGs in (**a**) CK-vs.-T_1_ and (**b**) CK-vs.-T_3_ comparison groups after 14 d of exposure to different concentrations of CuO NPs. The red color of the small square reflects the up-regulated DEGs, and the blue color of the small square reflects the down-regulated DEGs induced by the treatment compared with the control.

As shown in Figure 9a and Figure 10 of the top 20 enriched GO terms ranked by BH-adjusted *p* values in the CK-vs.-T_1_ comparison belonged to the biological process (BP) category. These BP terms included xylulose metabolic process (GO:0005997) and D-xylose metabolic process (GO:0042732), highlighting the enrichment of carbohydrate-related biological processes. Other top-ranked terms included molecular function (MF) terms related to calcium ion binding (GO:0005509), D-xylulokinase activity (GO:0004856), and oxidoreductase activity (GO:0016491). The cellular component (CC) terms included intrinsic component of membrane (GO:0031224), membrane (GO:0016020), integral component of membrane (GO:0016021), and cell periphery (GO:0071944).

In the CK-vs.-T_3_ comparison (Figure 9b), 5 of the top 20 enriched GO terms belonged to BP, 5 to MF, and 10 to CC. The BP terms were mainly associated with cell wall modification, including cell wall modification involved in abscission (GO:0009830) and cell wall organization or biogenesis (GO:0071554). The MF terms included hydrolase activity, hydrolyzing O-glycosyl compounds (GO:0004553), hydrolase activity, acting on glycosyl bonds (GO:0016798), oxidoreductase activity (GO:0016491), oxidoreductase activity, acting on paired donors, with incorporation or reduction of molecular oxygen (GO:0016705), and tetrapyrrole binding (GO:0046906). These terms primarily represented hydrolytic and redox-related enzymatic functions, together with tetrapyrrole binding. The CC terms were mainly associated with the cell wall and membrane structures, including cell wall (GO:0005618), intrinsic component of membrane (GO:0031224), intrinsic component of plasma membrane (GO:0031226), and integral component of plasma membrane (GO:0005887). Other enriched CC terms included external encapsulating structure (GO:0030312), cell periphery (GO:0071944), apoplast (GO:0048046), and beta-galactosidase complex (GO:0009341).

In the T_1_-vs.-T_3_ comparison (Figure 9c), the enriched BP terms were primarily associated with cell wall modification and carbohydrate and polysaccharide metabolism and catabolism. These included cell wall modifications related to abscission (GO:0009830), cell wall modification (GO:0042545), plant-type cell wall modification (GO:0009827), carbohydrate metabolic process (GO:0005975), carbohydrate catabolic process (GO:0016052), polysaccharide metabolic process (GO:0005976), polysaccharide catabolic process (GO:0000272), and cell wall disassembly (GO:0044277). The enriched MF terms included calcium ion binding (GO:0005509) and carbohydrate transmembrane transporter activity (GO:0015144). Most DEGs associated with these MF terms were upregulated.

KEGG pathway enrichment analysis further revealed the pathways represented by DEGs in the three pairwise comparisons. As shown in Figure 9d,e, the top 20 enriched KEGG pathways included 247 DEGs in the CK-vs.-T_1_ comparison and 397 DEGs in the CK-vs.-T_3_ comparison. Metabolism-related pathways predominated in both comparisons. Four pathways were common to the top 20 enriched pathways in both comparisons: phenylpropanoid biosynthesis (ko00940), alpha-linolenic acid metabolism (ko00592), brassinosteroid biosynthesis (ko00905), and ABC transporters (ko02010).

In the CK-vs.-T_1_ comparison, 15 of the top 20 KEGG pathways were metabolism-related. Significantly enriched pathways included ascorbate and aldarate metabolism (ko00053), cysteine and methionine metabolism (ko00270), and glutathione metabolism (ko00480), for which the associated DEGs were predominantly downregulated. In the CK-vs.-T3 comparison, 17 of the top 20 pathways were metabolism-related. Significantly enriched pathways included photosynthesis—antenna proteins (ko00196), starch and sucrose metabolism (ko00500), and pentose and glucuronate interconversions (ko00040). The significant downregulation of DEGs associated with light-harvesting chlorophyll protein complexes (LHCs) in the photosynthesis—antenna proteins pathway (Figure S3) was consistent with the reduced leaf photosynthetic capacity observed in *P. stratiotes* (Figure 3).

In the T_1_-vs.-T_3_ comparison, 464 DEGs were represented among the top 20 enriched KEGG pathways (Figure 9f). Seventeen of these pathways were metabolism-related, including significantly enriched pathways associated with pentose and glucuronate interconversions (ko00040), flavonoid biosynthesis (ko00941), fatty acid degradation (ko00071), and tyrosine metabolism (ko00350). In addition, protein processing in the endoplasmic reticulum (ko04141), a pathway associated with genetic information processing, was significantly enriched in the CK-vs.-T_1_ comparison, with the associated DEGs predominantly downregulated. In contrast, DEGs associated with this pathway were predominantly upregulated in T_3_ relative to T_1_. A similar contrasting expression pattern across comparisons was observed for DEGs associated with flavonoid biosynthesis.

### 2.8. Integrated Analysis of Transcriptomic and Metabolomic Pathway Responses

To compare transcriptomic and metabolomic responses at the pathway level, KEGG enrichment results for DEGs and DAMs were compared for each treatment contrast. As shown in Table S4, the DEGs and DAMs enriched in KEGG pathways were analyzed for each comparison. In the CK-vs.-T_1_ comparison, a total of 16 KEGG pathways were identified, representing pathways shared between the transcriptomic and metabolomic enrichment results. Among them, the pathways classified under Metabolism included biotin metabolism (ko00780), linoleic acid metabolism (ko00591), cysteine and methionine metabolism (ko00270), fatty acid biosynthesis (ko00061), and carotenoid biosynthesis (ko00906). The other category was Environmental Information Processing, with only one pathway, namely ABC transporters (ko02010).

In the CK-vs.-T_3_ comparison, the identified KEGG pathways also included pathways shared between the two omics datasets. Among them, 28 KEGG pathways classified under Metabolism were identified, including starch and sucrose metabolism (ko00500), pentose and glucuronate interconversions (ko00040), alpha-linolenic acid metabolism (ko00592), glyoxylate and dicarboxylate metabolism (ko00630), phenylpropanoid biosynthesis (ko00940), and fatty acid degradation (ko00071). There were 2 pathways classified under Environmental Information Processing, including ABC transporters (ko02010) and plant hormone signal transduction (ko04075).

The shared KEGG pathways between the two comparisons included ABC transporters, carotenoid biosynthesis, lysine degradation (ko00310), nicotinate and nicotinamide metabolism (ko00760), pentose and glucuronate interconversions, porphyrin metabolism (ko00860), and tyrosine metabolism (ko00350). The recurrence of these pathways across both comparisons suggests that these metabolic processes represent key components of the transcriptional and metabolic responses to CuO NP exposure.

### 2.9. MapMan Metabolic Pathway Analysis

MapMan analysis showed that leaf DEGs following CuO NP exposure were mainly assigned to stress- and response-related functional categories, broadly consistent with the KEGG enrichment results. The “Pathogen attack” category shown in the MapMan overview is a functional classification within the MapMan framework and should not be interpreted as evidence of pathogen exposure or infection in this study. Within the Response category, DEGs were mainly associated with “signaling”, “secondary metabolism”, “proteolysis”, and “cell wall” (Figure 10; Table S5).

For signaling-related functions, the CK-vs.-T_1_ and CK-vs.-T_3_ comparisons contained 51 and 61 DEGs, respectively, with 49 (96.08%) and 40 (65.57%) downregulated and 2 (3.92%) and 21 (34.43%) upregulated. These DEGs mainly involved calcium signaling and receptor kinases, with additional changes involving G-proteins and MAP kinases in CK-vs.-T_3_, indicating broader changes in signaling-related components under the higher treatment concentration.

For the cell wall category, CK-vs.-T_1_ and CK-vs.-T_3_ contained 30 and 55 DEGs, respectively, with 29 (96.67%) and 34 (61.82%) downregulated and 1 (3.33%) and 21 (38.18%) upregulated. These DEGs mainly involved cellulose and hemicellulose synthesis, cell wall proteins, degradation, modification, and pectin esterases, suggesting changes in cell wall biosynthesis and remodeling.

The protein degradation category contained 23 and 54 DEGs in CK-vs.-T_1_ and CK-vs.-T_3_, respectively, with 15 (65.22%) and 29 (53.70%) downregulated and 8 (34.78%) and 25 (46.30%) upregulated. These DEGs mainly involved subtilases, AAA-type ATPases, E3 RING, E3 SCF FBOX, and aspartate proteases, with additional changes in cysteine and serine proteases and metalloproteases in CK-vs.-T_3_ comparison, indicating altered expression of genes involved in protein turnover.

For secondary metabolism, the CK-vs.-T_1_ and CK-vs.-T_3_ comparisons contained 25 and 38 DEGs, respectively, with 24 (96.00%) and 17 (44.74%) downregulated and 1 (4.00%) and 21 (55.26%) upregulated. These DEGs mainly involved phenylpropanoids, flavonoids, glucosinolates, isoprenoids/terpenoids, and wax, with predominantly downregulated expression in CK-vs.-T_1_ but a more balanced expression pattern in CK-vs.-T_3_.

### 2.10. RT-qPCR Validation

**Figure 11:**
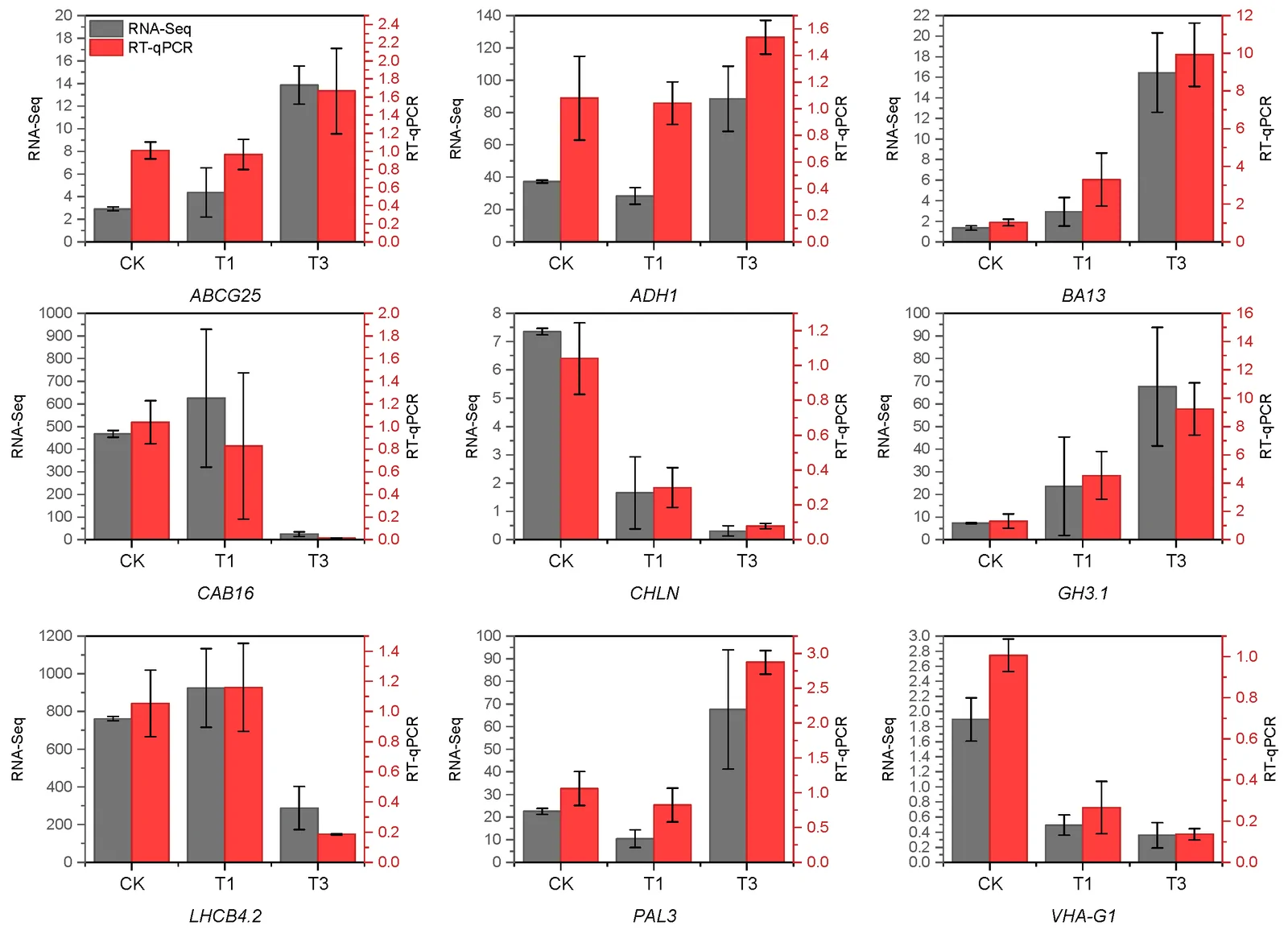
RT-qPCR verification of 9 selected DEGs after 14 d of CuO NP exposure. RT-qPCR results are compared with RNA-seq data to assess the consistency of gene expression patterns.

To validate the transcriptome sequencing data, quantitative real-time PCR (RT-qPCR) was performed for nine selected genes (*ABCG25*, *ADH1*, *BA13*, *CAB16*, *CHLN*, *GH3.1*, *LHCB4.2*, *PAL3*, and *VHA-G1*). Actin served as an internal control for normalization. As shown in Figure 11, the relative expression levels of all tested genes exhibited trends consistent with the RNA-Seq results, thereby confirming the reliability of the transcriptomic analysis.

## 3. Discussion

*Pistia stratiotes* has been studied as a candidate for aquatic phytoremediation because it can grow as a floating macrophyte and has been investigated for nutrient removal and the accumulation or handling of several metals [37,38,42,43,44]. Studies have examined plant-based remediation of mine tailings [46] and the use of invasive plants for potentially toxic-element accumulation [47], providing broader context for phytoremediation but not establishing the net remediation efficiency of *P. stratiotes* in CuO NP-contaminated water. The present study extends previous research by examining the physiological and elemental responses of *P. stratiotes* to different CuO NP exposure concentrations and durations and relating these responses to leaf transcriptomic and metabolomic profiles. Because phytoremediation performance depends on contaminant accumulation as well as the maintenance of plant growth and physiological function, both aspects must be evaluated. CuO NPs have attracted attention because of their potential environmental release and ecological effects [24,25]. Previous studies have shown that CuO NP exposure can induce oxidative stress and cellular damage in plants, potentially involving both particle-associated effects and the release of Cu ions [36]. Cell-wall components, particularly pectin, can bind metal ions, and changes in the pectin structure may influence metal retention and cellular function under metal stress [34,48,49,50]. These findings provide a basis for examining how Cu partitioning, physiological responses, and cell-wall-related processes are associated during CuO NP exposure in *P. stratiotes*.

Under low-concentration CuO NP exposure (T_1_), visible injury was limited, and leaf number did not differ significantly from CK. (Figure 1a). After 14 d, Chl a, Chl b, and Car contents increased by 12.73%, 10.54%, and 22.73%, respectively (Figure 3a–c), whereas *P*_n_, *C*_i_, and CE were not significantly affected (Figure 3e,f,h). Most fluorescence parameters, including φP_0_, also remained stable (Figure 4), suggesting that maximum PSII photochemical efficiency was largely maintained. The increased pigment contents therefore coincided with the maintenance of photosynthetic performance rather than a measurable increase in net photosynthesis. Cu is an essential micronutrient involved in photosynthetic electron transport [7] and adequate Cu can also participate in processes related to chlorophyll biosynthesis [51], while carotenoids can protect photosystems by quenching singlet oxygen and dissipating excess excitation energy [52]. The altered pigment profile may therefore have contributed to maintaining photosynthetic function under T_1_, particularly through changes in pigment-associated photoprotection.

APX activity increased significantly by 29.17% after 7 d, while DHAR activity increased by 2.08-fold and 5.30-fold after 7 and 14 d, respectively (Figure 2g,h). However, H_2_O_2_, O_2_·^−^, and MDA did not change significantly. The combination of increased APX and DHAR activities with stable ROS and MDA levels indicates that selected antioxidant responses changed without a detectable increase in the measured oxidative markers. CuO NPs and Cu ions released during dissolution may promote ROS generation through redox cycling and related reactions [53]. The unchanged ROS and MDA levels, together with the changes in APX and DHAR activities, are consistent with adjustments in antioxidant defense, although the contribution of individual defense pathways remains to be established. Similar responses to moderate nanoparticle exposure, including changes in photosynthetic pigments and antioxidant defenses, have been reported elsewhere [54]. Thus, the maintenance of photosynthetic performance at T_1_ did not prevent growth inhibition, indicating that root growth was more sensitive to this exposure than several aboveground photosynthetic parameters. The T_1_ response should consequently not be interpreted as evidence of confirmed hormesis.

Under higher-concentration CuO NP exposure (T_3_ and T_4_), *P. stratiotes* showed pronounced photosynthetic inhibition. After 7 and 14 d, *P*_n_ and CE decreased significantly, with reductions ranging from 42.62% to 66.67%, whereas *C*_i_ increased by 8.20–14.43% (Figure 3e,f,h). The decrease in *P*_n_ accompanied by an increase in *C*_i_ suggests that stomatal limitation alone may not fully explain the inhibition of photosynthesis, and that mesophyll or biochemical limitations may also have contributed [55]. Chlorophyll fluorescence responses were consistent with this interpretation. After 14 d, *F*_m_, φP_0_, φE_0_, and PI_ABS_ decreased significantly by 15.57–75.12% in the T_3_ and T_4_, whereas φD_0_, ABS/RC, and DI_0_/RC increased significantly by 29.28–146.00% (Figure 4). These changes indicate impaired PSII photochemistry and electron transport, accompanied by increased energy dissipation under higher-concentration exposure [56].

O_2_·^−^ contents increased significantly by 73.30–349.47% in T_3_ and T_4_ (Figure 2d). The activities of POD, CAT, APX, DHAR, and GR also increased (Figure 2e–i), accompanied by increases in GSH and Cys concentrations of 17.21–35.94% and 25.00–52.78%, respectively (Figure 2k,i). GSH and sulfur-containing metabolites play important roles in the detoxification of heavy metals and ROS [33,57]. The concurrent increases in O_2_·^−^, antioxidant enzyme activities, GSH, and Cys indicate that higher-concentration exposure elicited a substantial redox and thiol-associated response alongside photosynthetic impairment. These responses were not sufficient to maintain photosynthetic performance. Notably, MDA content decreased after 14 d in T_2_–T_4_, despite significant increases in O_2_·^−^ in T_2_ and T_4_. Because MDA reflects lipid peroxidation rather than ROS abundance itself, the two indicators did not change in parallel. This divergence highlights the importance of distinguishing ROS accumulation from the extent of lipid peroxidation when evaluating oxidative responses to CuO NP exposure.

Root Cu concentrations increased significantly in all CuO NP treatments, whereas TF decreased by 76.92–86.15% (Table 1). Because the tissues were acid-digested before ICP-MS analysis, the measurements represent total tissue Cu and cannot distinguish between Cu associated with intact CuO NPs and Cu derived from dissolved species. BCF increased significantly by 301.93% in T_1_, did not differ significantly from CK in T_2_, and decreased by 61.65% and 73.69% in T_3_ and T_4_, respectively (Table 2). Thus, root Cu accumulation increased across treatments, but accumulation relative to the nominal total Cu concentration in the exposure solution declined at higher concentrations. The increase in root Cu concentrations, coupled with the decline in TF, indicates preferential Cu retention in roots and reduced translocation to shoots, potentially limiting Cu delivery to photosynthetic tissues [58]. Such root-biased Cu distribution may involve changes in Cu uptake and intracellular or apoplastic metal complexation [48,59], although these processes were not directly measured.

CuO NP exposure also altered the distribution of several mineral nutrients. Leaf Fe and root Ca remained largely stable, whereas root Zn decreased under T_1_ and T_2_ but increased under T_4_ (Figure S3). More notably, leaf S increased by 20.89–37.56% across the CuO NP treatments. At the higher concentrations (T_3_ and T_4_), the increase in leaf S was accompanied by marked increases in leaf Cu, Cys, and GSH (Table 2; Figure 2i,k and Figure S3). This coordinated pattern is consistent with the broader sulfur-associated response reflected by the increases in Cys and GSH, rather than representing an independent change in leaf S. Sulfur is required for Cys biosynthesis, and Cys is a precursor of GSH. Cys provides thiol groups that can participate in Cu binding and is also a precursor of GSH. The thiol groups of Cys can participate in Cu binding, while GSH contributes to both antioxidant defense and metal handling [57,60]. The coordinated increases in Cu, S, Cys, and GSH therefore suggest that sulfur- and thiol-associated metabolism is involved in the response to elevated Cu burden, potentially contributing to antioxidant defense and Cu complexation. Although Cu–thiol complexes and their subcellular localization were not directly measured, this coordinated response provides a plausible biochemical link between increased Cu accumulation and changes in sulfur metabolism.

The transcriptomic and metabolomic analyses provide further insight into the molecular adjustments underlying the physiological responses observed at T_1_ and T_3_. These analyses were restricted to leaf samples from CK, T_1_, and T_3_ collected after 14 d of exposure. In the CK-vs.-T_1_ comparison, 588 DEGs were identified, including 79 upregulated and 509 downregulated genes in T_1_ relative to CK (Figure S1a), together with 166 DAMs. KEGG enrichment analysis of the DAMs highlighted carotenoid biosynthesis, cutin, suberin, and wax biosynthesis, purine metabolism, and histidine metabolism (Figure 7a). Although photosynthetic performance was largely maintained at T_1_, these transcriptomic and metabolomic changes, together with the decreases in the root dry weight, RGR, and STI, indicate that this apparent physiological stability was accompanied by substantial molecular adjustment and growth costs.

In the CK-vs.-T_3_ comparison, 1088 DEGs were identified, including 542 upregulated and 546 downregulated genes in T3 relative to CK (Figure S1a), together with 352 DAMs. The T_1_-vs.-T_3_ comparison identified 844 DEGs, including 728 upregulated and 116 downregulated genes in T_3_ relative to T_1_, together with 240 DAMs. The DAMs were associated with a broader range of metabolic pathways, including carotenoid biosynthesis, plant hormone-related metabolism, amino acid degradation, and glucosinolate metabolism (Figure 7b,c). The larger numbers of DEGs and DAMs in CK-vs.-T_3_ than in CK-vs.-T_1_, together with the broader range of affected pathways, corresponded to the stronger physiological stress at T_3_, characterized by reduced *P*_n_ and CE, impaired PSII photochemistry, and increased O_2_·^−^ accumulation. These patterns indicate a transition from molecular adjustment under T_1_ to a broader transcriptional and metabolic response under T_3_.

Genes encoding light-harvesting complexes (LHCs) were downregulated after T_3_ exposure relative to CK (Figure S2). LHC proteins mediate light harvesting and transfer excitation energy to PSII [61]. The downregulation of LHC-related genes provides a molecular correlate of the reduced *P*_n_ and CE and the decreases in φP_0_, φE_0_, and PI_ABS_ observed under T_3_ (Figure 3 and Figure 4), linking changes in light-harvesting components with impaired photosynthetic energy conversion. Previous studies have also shown that excess Cu can affect LHCII and PSII electron transport, thereby impairing photosynthetic function [7,62]. At the same time, several carotenoid metabolites accumulated under T_3_. Because carotenoids participate in excess-energy dissipation and ROS quenching [52], their accumulation may represent a compensatory metabolic adjustment accompanying the impairment of photosynthetic function. However, the direct photoprotective contribution of these carotenoids was not tested. The parallel changes in LHC-related transcripts, photosynthetic parameters, and carotenoid metabolites indicate that photosynthetic impairment under T_3_ coincided with substantial changes in pigment-associated metabolism.

**Figure 12:**
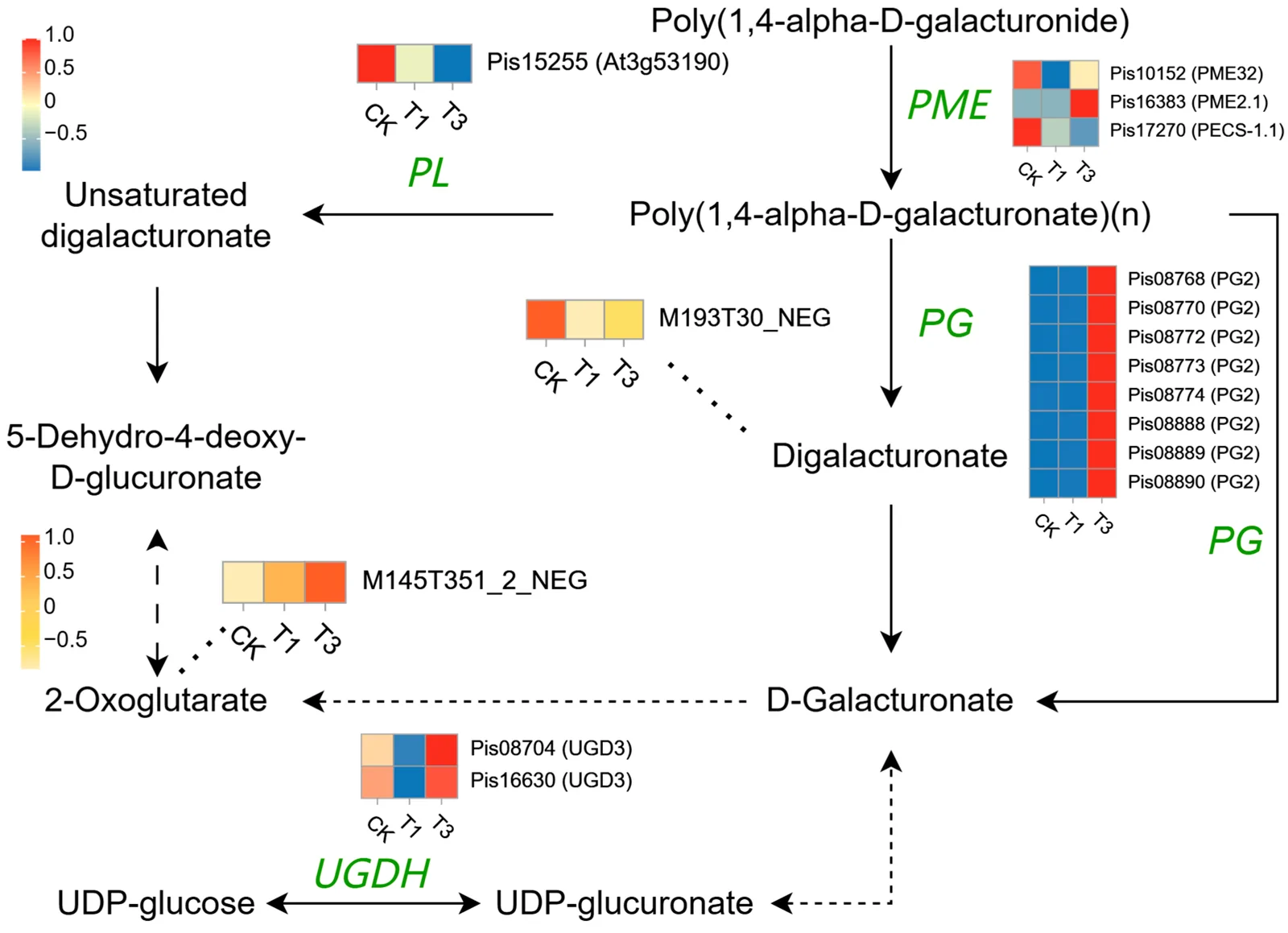
Coordinated transcriptomic and metabolomic changes associated with the pentose and glucuronate interconversions pathway after 14 d of CuO NP exposure (T_1_ and T_3_). Bidirectional arrows denote reversible reactions, whereas dashed arrows indicate indirect connections with intermediate steps omitted. *PL*, pectate lyase; *PME*, pectinesterase; *PG*, polygalacturonase; *UGDH*, UDP-glucose 6-dehydrogenase; *UGD3, UDP-glucose 6-dehydrogenase 3*.

Cell wall remodeling is an important response to metal stress, with pectin playing a key role because its negatively charged groups can bind metal ions and its degree of methylesterification can influence cell wall properties and metal binding [49,50]. CuO NPs have also been reported to alter cell wall polysaccharides and pectin, potentially affecting root growth and Cu retention [34]. In line with these previous findings, our transcriptomic and metabolomic analyses revealed treatment-related changes in the pentose and glucuronate interconversions pathway in *P. stratiotes* to CuO NPs (Figure 12). Several genes in this pathway are associated with cell wall polysaccharide synthesis and pectin modification. *UGD3* (UDP-glucose 6-dehydrogenase 3) is involved in UDP-glucuronic acid-related metabolism and provides precursors for cell wall polysaccharide biosynthesis, whereas *PME32* (pectin methylesterase 32) and *PECS-1.1* (pectinesterase) are involved in pectin modification. Polygalacturonase (PG) family enzymes contribute to the degradation of de-esterified pectin and cell wall remodeling [63,64].

Under low-concentration exposure (T_1_), *UGD3*, *PME32*, and *PECS-1.1* were downregulated, accompanied by a decrease in digalacturonic acid abundance. In contrast, under high-concentration exposure (T_3_), *PME2.1* and eight PG2 family genes were upregulated, whereas *PECS-1.1* and the pectate lyase gene *Pis15255* were downregulated, indicating distinct patterns of expression among genes associated with pectin modification and degradation under the two exposure conditions. These contrasting expression patterns indicate concentration-dependent changes in pectin-related metabolism rather than uniform activation or repression of the pathway. The changes occurred alongside the observed shift toward greater root Cu accumulation and reduced translocation to shoots. Because pectin contains negatively charged groups capable of binding metal ions [48,49,65], remodeling of pectin-rich cell-wall components could alter the capacity of roots to retain Cu and thereby influence Cu partitioning between roots and leaves. The concurrent changes in pectin-related gene expression and Cu distribution support a potential role for cell-wall remodeling in root Cu retention and reduced Cu translocation. Although cell-wall composition, pectin methylesterification, and cell-wall-bound Cu were not directly measured, these findings identify pectin-related remodeling as a plausible link between CuO NP exposure and altered Cu partitioning.

The different expression patterns of NAC, WRKY, and ERF transcription factors further support the differential regulation of pectin metabolism under T_1_ and T_3_. These transcription factors were generally downregulated under T_1_, whereas most NAC genes and several WRKY and ERF genes were upregulated under T_3_ (Figure S4). This shift toward increased expression of several stress-responsive transcription factors at T_3_ is consistent with the broader transcriptional response and altered cell-wall-related gene expression at the higher concentration [66,67]. In addition, 2-oxoglutarate abundance increased significantly under T_3_, potentially reflecting changes in central carbon metabolism and the redistribution of carbon skeletons under stress [68]. The expression shifts in NAC, WRKY, and ERF genes, along with the increased abundance of 2-oxoglutarate under T_3_, point to changes in stress-responsive transcriptional regulation and central carbon metabolism alongside cell-wall-related responses.

**Figure 13:**
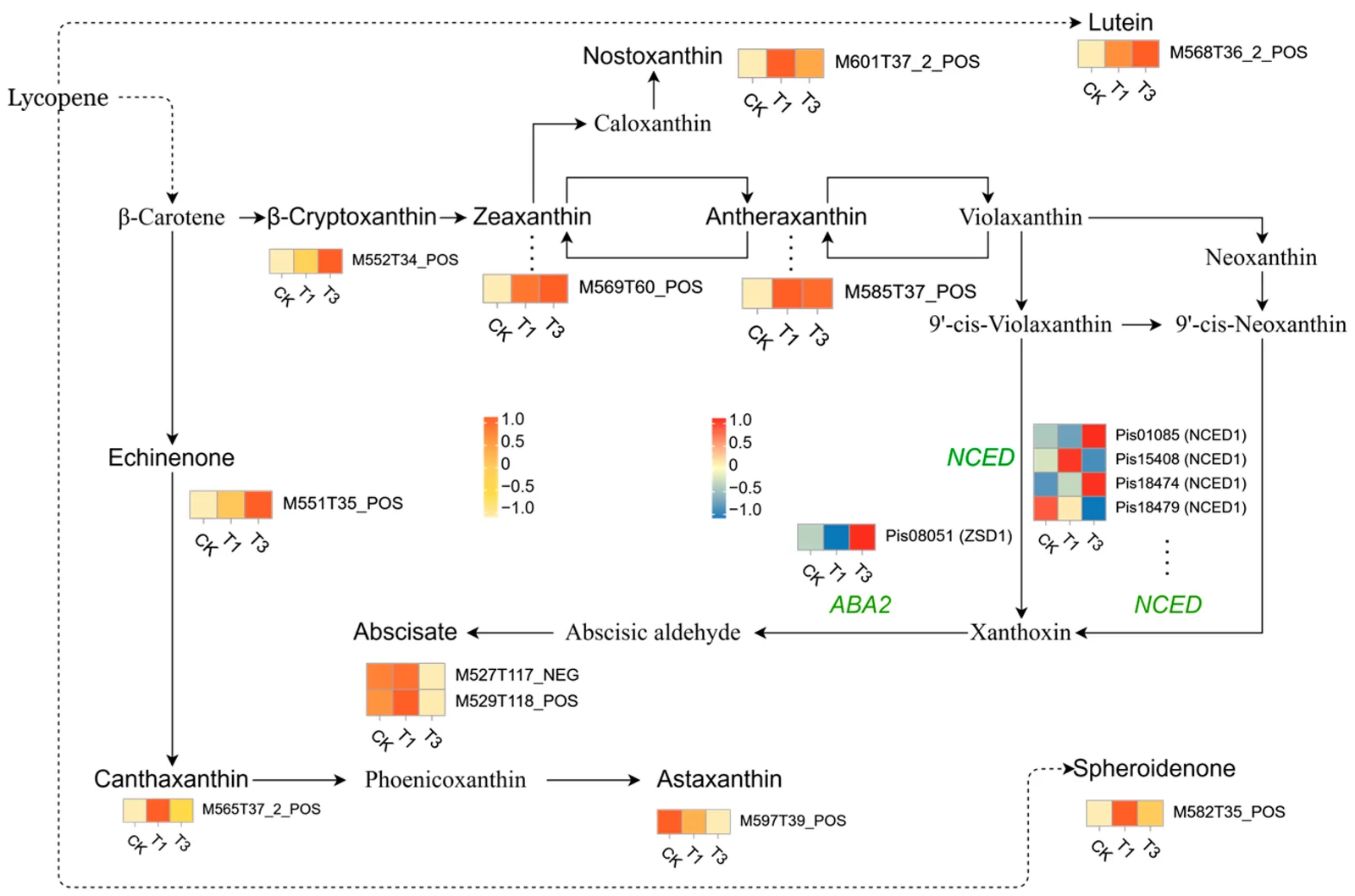
Coordinated transcriptomic and metabolomic changes associated with the carotenoid biosynthesis pathway after 14 d of CuO NP exposure (T_1_ and T_3_). Dashed arrows indicate indirect connections with intermediate steps omitted. *NCED*, 9-cis-epoxycarotenoid dioxygenase; *ABA2*, xanthoxin dehydrogenase.

Changes in the carotenoid biosynthesis pathway at both the transcript and metabolite levels suggested that carotenoid metabolism was involved in the response of *P. stratiotes* to CuO NP exposure. Carotenoids are important components of the photosynthetic system and precursors of plant hormones such as abscisic acid (ABA), and their changes can contribute to photoprotection and stress responses [69]. As shown in Figure 13, the abundances of zeaxanthin, antheraxanthin, canthaxanthin, β-cryptoxanthin, β-echinenone, lutein, and nostoxanthin were significantly increased in T_1_, indicating changes in the carotenoid metabolite profile under low-concentration CuO NP exposure. Zeaxanthin and lutein are associated with PSII photoprotection and the dissipation of excess excitation energy [52,70]. The increased abundance of several carotenoids, together with the largely stable photosynthetic performance under T_1_, is consistent with a role for carotenoid-related metabolic adjustments in the response to low-concentration exposure.

The carotenoid-to-ABA branch was also differentially regulated. 9-cis-epoxycarotenoid dioxygenase (NCED) genes catalyze the cleavage of 9-cis-epoxycarotenoids, an important step in ABA biosynthesis, whereas xanthoxin dehydrogenase (*ABA2*/*ZSD1*) catalyzes the subsequent conversion of xanthoxin to abscisic aldehyde [71,72]. Under T_1_, *Pis15408* (*NCED1*) was upregulated, whereas *Pis01085* (*NCED1*) and *Pis0805*1 (*ZSD1*/*ABA2*) were downregulated. ABA abundance, however, did not change significantly. Thus, the mixed expression patterns of ABA-related genes were not accompanied by a detectable change in the ABA pool under T_1_. Under T_3_, the abundances of zeaxanthin, antheraxanthin, β-cryptoxanthin, β-echinenone, lutein, spheroidenone, and nostoxanthin remained significantly higher, whereas those of astaxanthin, 2-cis-4-trans-abscisic acid, and (+)-abscisic acid were significantly lower. At the same time, *Pis18479* (*NCED1*) was downregulated, whereas *Pis01085* (*NCED1*) and *Pis18474* (*NCED1*) were upregulated, indicating differential regulation of carotenoid- and ABA-related profiles under higher-concentration CuO NP exposure. Changes in the ABA branch mediated by *NCED* genes can be accompanied by changes in carotenoid composition. For example, suppression of tomato *SlNCED1* decreased ABA levels and was accompanied by lycopene and β-carotene accumulation [73]. In the present study, the contrasting expression patterns of NCED-related genes and changes in ABA-associated metabolites, alongside the accumulation of several carotenoids, point to distinct responses of carotenoid metabolism and the ABA-related branch under T_1_ and T_3_ rather than uniform regulation of the entire pathway. These findings associate carotenoid- and ABA-related metabolic changes with the contrasting physiological responses observed under T_1_ and T_3_. Under T_1_, increased abundances of several carotenoids coincided with largely maintained photosynthetic performance, whereas under T_3_, changes in carotenoid and ABA-related metabolites accompanied reductions in *P*_n_ and CE, impaired PSII photochemistry, and increased O_2_·^−^ accumulation (Figure 2, Figure 3 and Figure 4).

Overall, the physiological, elemental, transcriptomic, and metabolomic results indicate that *P. stratiotes* responded to CuO NP exposure through coordinated changes in Cu partitioning, photosynthetic function, redox homeostasis, and cellular metabolism. At T_1_, largely stable photosynthetic performance was accompanied by increased photosynthetic pigment contents and changes in carotenoid metabolism and antioxidant-related parameters, despite measurable reductions in root biomass and growth-related indices. At T_3_, the higher Cu burden was associated with impaired photosynthetic energy conversion, increased O_2_·^−^ accumulation, and a broader transcriptional and metabolic response, including changes in LHC-related genes, carotenoid metabolism, and stress-related pathways. The increased root Cu concentration and reduced TF across treatments, together with concentration-dependent changes in pectin-related genes, further suggest a potential link between Cu partitioning and cell-wall-related processes. The results support a multi-level response in which Cu accumulation and partitioning were accompanied by adjustments in photosynthetic processes, antioxidant defenses, cell-wall-related metabolism, and carotenoid- and hormone-associated pathways. The response to CuO NP exposure was therefore concentration dependent, with broader physiological and molecular perturbations at higher concentrations. Because dissolved Cu concentrations were not measured and no soluble-Cu control was included, these findings should be interpreted as responses to CuO NP exposure as a whole rather than effects attributable exclusively to intact nanoparticles.

## 4. Materials and Methods

### 4.1. CuO NP Treatment and Stress Injury Assessment

Seedlings of *Pistia stratiotes* from the current year were purchased from Zhangpu Town, Kunshan City, Jiangsu Province, China. After transport-related losses were removed, the plants were cultivated outdoors in tap water to allow recovery and resumed growth. Before exposure, uniformly growing plants were selected, all daughter plants were removed, and five mature leaves were retained on each mother plant. Plants were acclimated for 1 week in a transparent plastic shelter in plastic containers (internal dimensions, 550 × 410 × 190 mm) containing 20 L of half-strength Hoagland nutrient solution. The exposure experiment was then conducted in the same type of container, with 20 L of half-strength Hoagland solution per container and a pH of 6.5 ± 0.1 throughout the experiment.

CuO NPs (Product No. C805376; CAS No. 1317-38-0; purity, 99.5%; nominal particle size, 40 nm; spherical; D50, 30–50 nm; Shanghai Macklin Biochemical Co., Ltd., Shanghai, China) were used. According to the manufacturer, the material’s crystal structure had been confirmed by X-ray diffraction. The experiment included an untreated control (CK; no CuO NPs added) and four CuO NP-amended treatments (T_1_–T_4_), corresponding to nominal Cu concentrations of 0.1, 1.0, 5.0, and 15.0 mg·L^−1^, respectively. Cu was supplied in the form of CuO NPs, and the concentrations were expressed on an elemental-Cu basis. These concentrations corresponded to 0.1 ×, 1 ×, 5 ×, and 15 × the maximum Cu specified in China’s Environmental Quality Standards for Surface Water (GB 3838–2002) [74], which also expresses the Cu·Limit on an elemental-Cu basis. The required mass of CuO NPs was calculated from the theoretical Cu mass fraction in CuO (63.546/79.545) and adjusted for the manufacturer-reported purity of 99.5%. A CuO NP stock suspension was then prepared at 5 mg·mL^−1^ by magnetic stirring for 30 min. Before each addition to an exposure system, the stock suspension was manually stirred again to resuspend settled particles, and the required volume was immediately transferred using a micropipette. Each exposure system contained a final volume of 20 L of 1/2-strength Hoagland solution. To obtain the target nominal Cu concentrations, 0.503, 5.032, 25.16, and 75.47 mL of the 5 mg·mL^−1^ CuO NP stock suspension were added to T_1_, T_2_, T_3_, and T_4_, respectively. The stock suspension was re-stirred before each transfer to minimize particle settling during preparation and addition, whereas CK received no CuO NP suspension. During the 14-day exposure period, the solutions were neither renewed nor continuously stirred. The solutions were not renewed to avoid repeatedly removing the plants from the containers, which could have disturbed the plants during exposure. Actual Cu concentrations in the exposure solutions were not measured.

Three independent containers were used per treatment, and 12 plants were placed in each container. Thus, the container was the experimental unit *(n* = 3 containers per treatment); plants within a container were treated as subsamples rather than independent exposure replicates. The exposure lasted 14 d. Leaf number and injury scores were recorded at 0, 7, and 14 d, and the injury index was calculated as described below. After washing, fresh samples were stored at −80 °C until analysis. Wastewater containing CuO NPs was collected and sent to Jiangsu Environmental Resources Co., Ltd. (Nanjing, China) for appropriate treatment. The injury index was calculated using a weighted injury-score method, as described in previous studies of plant stress responses [75], as follows:

Injury Index = Σ (Injury score × Number of plants at each injury score)/(Total number of plants × Maximum injury score)

Note: If all plants in a treatment group were classified as grade 0, the injury index was assigned a value of 0.

### 4.2. Biomass Parameters and Cu Content Determination

Plants collected at 0 and 14 d were immersed in 20 mmol·L^−1^ Na_2_-EDTA solution for 20 min and rinsed thoroughly with ultrapure water to remove surface-associated Cu [76,77]. Because chelator-based washing may also remove exchangeable or apoplast-associated metals and no washing-recovery test was conducted, the measured values represent total Cu concentrations in acid-digested, EDTA-washed tissues rather than intracellular Cu alone [76,78]. Residual water was removed with filter paper, and the plants were separated into leaves and roots. Samples were heated at 105 °C for 30 min and then dried at 80 °C to a constant weight. After accurately measuring the dry weight, the stress tolerance index (STI) [79] and relative growth rate (RGR) [80] were calculated as shown below. Dried leaf and root samples were ground and passed through a 100-mesh sieve. A 0.5 g subsample was digested with 5 mL of a mixed HNO_3_–HClO_4_ acid solution (4:1, *v*:*v*), left sealed at room temperature in the dark for 8 h, and heated at 220 °C until the digest became clear. After cooling, the digest was rinsed three times with ultrapure water and brought to 50 mL. The solution was filtered through a 0.22 μm aqueous syringe filter and analyzed by ICP-MS (NexION 300, PerkinElmer, Waltham, MA, USA). The Cu translocation factor (TF) and bioconcentration factor (BCF) of *P. stratiotes* were calculated according to the methods of Zabergja–Ferati et al. and Meng et al. [81,82].

Stress Tolerance Index (STI, %) = DW/DW_CK_ × 100%

Relative Growth Rate (RGR, mg/g/d) = [ln(DW) − ln(DW_0_)]/(t − t_0_)

where DW is the dry weight of each plant component at 14 d, DW_CK_ is the mean dry weight of each plant component in the control group at 14 d, DW_0_ is the mean dry weight of each plant component at 0 d, and t − t_0_ represents the entire 14 d treatment period.

Cu bioconcentration factor (BCF) = *C*_root_/*C*_Cu, nomina_l; *C*_Cu, nomina_l = *C*_Cu, background_+ *C*_Cu, added_

Cu translocation factor (TF) = *C*_leaf_/*C*_root_

where *C*_root_ and *C*_leaf_ are the total Cu concentrations in roots and leaves, respectively (mg·kg^−1^ DW); *C*_Cu, nomina_l is the nominal total Cu concentration in the exposure solution (mg Cu·L^−1^); *C*_Cu, background_ is is the nominal background Cu concentration in the half-strength Hoagland nutrient solution, based on its formulation; *C*_Cu, added_ is the nominal Cu concentration introduced in the form of CuO NPs (mg Cu·L^−1^). BCF is expressed in L kg^−1^ DW, whereas TF is dimensionless.

### 4.3. Determination of Antioxidant System and Ascorbate–Glutathione Cycle Parameters

Fresh leaf samples collected at 0, 7, and 14 d were used to determine the contents of soluble sugars, malondialdehyde (MDA), and reactive oxygen species (ROS), as well as the activities of antioxidant enzymes and the contents of non-enzymatic antioxidants and cysteine. The determination methods were based on previously established methods with minor modifications; detailed procedures are provided in the Appendix A.

### 4.4. Determination of Photosynthetic Pigment Contents, Gas Exchange Parameters, and Chlorophyll a Fluorescence Parameters

Fresh leaf samples (0.2 g) collected at 0, 7, and 14 d were placed in 20 mL of a 1:1 (*v*:*v*) mixture of acetone and absolute ethanol. After sealing, the samples were extracted at room temperature in the dark until the leaf tissues became completely white (approximately 48 h). The contents of chlorophyll and carotenoids were calculated by measuring the absorbance of the extracts at 665, 649, and 470 nm [83].

Gas exchange parameters, including net photosynthetic rate (*P*_n_), transpiration rate (*T*_r_), and intercellular CO_2_ concentration (*C*_i_), were measured between 9:00 and 11:00 at 0, 7, and 14 d using a CIRAS-3 portable photosynthesis–fluorescence system (PP Systems International, Inc., Amesbury, MA, USA). The third or fourth fully expanded functional leaf from the top of each plant was selected for measurement. The environmental conditions of the leaf chamber were set as follows: photosynthetically active radiation (PAR), 1200 μmol/(m^2^·s); leaf chamber temperature, 25.0 °C. Ambient air was supplied externally, and the CO_2_ concentration was maintained at 380–420 μmol/mol. Stomatal limitation (*L*_s_) was calculated as *L*_s_ = 1 − *C*_i_/*C*_a_ [84], where C_a_ is the ambient CO_2_ concentration. The instantaneous carboxylation efficiency (CE) was calculated as CE = *P*_n_/*C*_i_ [85].

After gas exchange measurements, the selected leaves were dark-adapted for 30 min, and chlorophyll a fluorescence parameters were measured using a Handy PEA+ continuous excitation fluorometer (Hansatech Instruments Ltd., King’s Lynn, Norfolk, UK). A saturating pulse of ultra-bright red light (650 nm, 3000 μmol/(m^2^·s)) was uniformly applied for 2 s to the uncovered leaf area within the leaf clip (a circular area with a diameter of 4 mm). The parameters derived from the OJIP transient were calculated using the JIP-test equations [86,87].

### 4.5. Metabolomics Analysis

Leaf samples (approximately 80 mg per sample) were collected from *P. stratiotes* plants after 14 d of exposure to CuO NPs at the T_1_ and T_3_ concentrations and from untreated control plants (CK), with three biological replicates per group. CK, T_1_, and T_3_ were selected for transcriptomic and metabolomic analyses to compare the control with a low-concentration treatment under which photosynthetic performance was largely maintained and a higher-concentration treatment associated with marked photosynthetic inhibition and other physiological changes. T_2_ and T_4_ were not included in the omics analyses; therefore, the molecular findings reflect responses under the selected exposure conditions rather than across the full concentration range.

Samples were frozen in liquid nitrogen and pulverized using a mortar and pestle. For metabolite extraction, 1000 μL of methanol:acetonitrile:water (2:2:1, *v*:*v*:*v*) was added to each homogenized sample. The mixture was centrifuged at 14,000× *g* for 15 min at 4 °C. The supernatant was collected and dried under vacuum, and the resulting extracts were reconstituted in 100 μL of acetonitrile:water (1:1, *v*:*v*) for LC–MS analysis. Metabolomic profiling was performed by Guangzhou Gene Denovo Biotechnology Co., Ltd. (Guangzhou, China) using an Agilent 1290 Infinity LC system (Agilent Technologies, Waldbronn, Germany) coupled to a TripleTOF 6600 quadrupole time-of-flight mass spectrometer (AB SCIEX, Framingham, MA, USA).

Raw MS data were converted to mzML format using ProteoWizard MSConvert [88] and processed using XCMS for peak detection, retention-time correction, peak grouping, and peak-area extraction [89]. Peak detection was performed using the centWave algorithm with [90] the following parameters: ppm = 25, peakwidth = c(10, 60), and prefilter = c(10, 100). Peak grouping was performed with bw = 5, mzwid = 0.025, and minfrac = 0.5. CAMERA was used for isotope and adduct annotation [91]. Features with more than 50% missing values were removed, and the remaining missing values were imputed using the k-nearest neighbors (KNN) method. Features with a relative standard deviation (RSD) greater than 50% were excluded. Peak-area data were normalized by total peak area separately for the positive- and negative-ionization mode datasets before downstream statistical analyses [92].

Metabolite annotation was based on accurate *m/z* matching (mass error < 10 ppm) and MS/MS spectral matching against public databases, including MassBank, METLIN, and MoNA, as well as an in-house MS/MS database containing reference spectra from available authentic standards. The available analytical report did not specify formal identification-confidence levels for individual annotations; therefore, metabolite annotations were described according to the reported matching procedures without assigning unverified confidence levels [93]. Pooled quality-control (QC) samples were prepared by mixing equal volumes of the study samples and inserted into the analytical sequence to monitor system stability. Principal component analysis (PCA) was performed in R using the gmodels package (version 2.18.1) to assess the distribution of the QC samples. The QC samples clustered closely in the PCA score plot, indicating good consistency among QC measurements.

Orthogonal partial least squares discriminant analysis (OPLS-DA) was performed using the ropls package in R [94]. Model performance was evaluated by seven-fold cross-validation and 200 permutation tests. The observed *R*^2^ and *Q*^2^ values were compared with those obtained from the permuted models to assess model fit and predictive performance. Differentially accumulated metabolites (DAMs) were identified based on two criteria: a nominal *p* value < 0.05 from Student’s *t*-test and a variable importance in projection (VIP) score ≥ 1.0 derived from the OPLS-DA model. Because the feature-level *p* values were not adjusted for multiple testing, the resulting DAMs were treated as exploratory candidates, and the possibility of false-positive findings was acknowledged. DAMs identified in the positive- and negative-ionization modes were subsequently combined for pathway-level analyses. KEGG pathway enrichment analysis of DAMs was performed using the hypergeometric test, with all detected metabolites annotated to KEGG serving as the background set [95]. The resulting *p* values were adjusted for multiple testing using the Benjamini–Hochberg (BH) procedure [96]. Pathways with BH-adjusted *p* values ≤ 0.05 were considered significantly enriched.

### 4.6. Transcriptome Analysis

For total RNA extraction and transcriptome sequencing, fresh leaves (0.5 g per replicate) were collected after 14 d of treatment from both CuO NPs-treated groups (T_1_ and T_3_) and the untreated control group (CK), with three biological replicates per group. Total RNA was isolated using the Trizol reagent kit (Invitrogen, Carlsbad, CA, USA), and its quality was evaluated with an Agilent 2100 Bioanalyzer (Agilent Technologies, Palo Alto, CA, USA) and RNase-free agarose gel electrophoresis. Eukaryotic mRNA was enriched with Oligo(dT) beads, fragmented, and reverse-transcribed into cDNA using an NEB Next Ultra RNA Library Prep Kit for Illumina (NEB #7530, New England Biolabs, Ipswich, MA, USA). After purification, end-repair, A-tailing, and adaptor ligation, the products were size-selected with AMPure XP Beads (1.0X) and PCR-amplified to construct the sequencing library. The libraries were sequenced on an Illumina NovaSeq 6000 platform by Gene Denovo Biotechnology Co., Ltd. (Guangzhou, China).

Sequencing quality was assessed using Q20, Q30, and GC content. Across the nine samples, Q20 values were ≥97.74%, Q30 values were ≥93.37%, and GC contents ranged from 49.78% to 50.83%. Pearson correlation coefficients among samples were ≥0.73. Clean reads were mapped to the *P. stratiotes* genome [97] using HISAT2 [98]. The total mapping rate ranged from 87.44% to 89.26%, with 84.42–87.08% of reads uniquely mapped, 2.16–3.02% mapped to multiple genomic locations, and 10.74–12.56% unmapped. Detailed sample-level mapping statistics are provided in Table S3.

Gene-level read counts were analyzed using DESeq2 (version 1.24) [99]. Read counts were normalized within DESeq2, and differential expression was assessed using its model-based statistical framework. The resulting *p* values were adjusted for multiple testing to control the false discovery rate (FDR). Genes with FDR < 0.05 and |log_2_ fold change| > 1 (equivalent to a fold change > 2 or <0.5) were considered differentially expressed genes (DEGs). The identified DEGs were functionally annotated using the NR, GO, and KEGG databases [95,100,101]. GO enrichment analysis was performed using the hypergeometric test, with all genes annotated to GO in the reference genome serving as the background set [102]. The resulting *p* values were adjusted for multiple testing using the Benjamini–Hochberg (BH) procedure [96], and GO terms with BH-adjusted *p* values ≤ 0.05 were considered significantly enriched. KEGG pathway enrichment analysis was performed using the hypergeometric test, with all genes annotated to KEGG in the reference genome serving as the background set. The resulting *p* values were adjusted for multiple testing using the BH procedure, and pathways with BH-adjusted *p* values ≤ 0.05 were considered significantly enriched. To compare the transcriptomic and metabolomic results, significantly enriched KEGG pathways from the two datasets were compared to identify shared pathways. The enrichment analyses were conducted independently using hypergeometric tests and the corresponding background sets, with BH-adjusted *p* values ≤ 0.05 used as the significance threshold for each analysis. Shared pathways were interpreted as associations between transcriptional and metabolic responses rather than evidence of direct gene–metabolite interactions or causal relationships.

For qPCR validation, total RNA was re-extracted using the SteadyPure Plant RNA Extraction Kit (Accurate, Changsha, China), and cDNA was synthesized with the Evo M-MLV RT Mix Kit (Accurate, Changsha, China). Specific primers were designed with the NCBI Primer-BLAST tool (www.ncbi.nlm.nih.gov/tools/primer-blast, accessed on 26 December 2024) [103] (Table S6). RT-qPCR was subsequently performed using the SYBR Green Pro Taq HS Premixed qPCR Kit (Accurate, Changsha, China) on a StepOnePlus Real-Time PCR System (Waltham, MA, USA), and relative expression levels were determined by the 2^−ΔΔCT^ method [104] with three independent biological replicates. MapMan was used to visualize DEGs associated with metabolic pathways and other functional categories [105,106]. The RNA-seq data have been deposited in the NCBI SRA under BioProject accession number PRJNA1544582, SRA Study accession number SRP746537, and run accession numbers SRR41225141–SRR41225149.

### 4.7. Statistical Analysis

Physiological data were analyzed using SPSS Statistics (version 27.0). At each sampling time (7 and 14 d), treatment differences were assessed separately using one-way analysis of variance (ANOVA), with the CuO NP concentration as the factor. Concentration-by-time interactions were not tested because the data from the two sampling times were analyzed separately. Three independent containers were used per treatment, with 12 plants in each container. Each container served as one biological replicate (*n* = 3 per treatment), and the data from each container were used as one independent observation for each physiological variable. Plants within the same container were treated as subsamples rather than independent replicates. Different individual plants were sampled at 7 and 14 d; therefore, the same plants were not measured repeatedly. Data normality was assessed using the Shapiro–Wilk test, and homogeneity of variances was assessed using Levene’s test. When significant treatment effects were detected, Duncan’s multiple range test was used for post hoc comparisons. Values are presented as means ± standard error (SE), and differences were considered statistically significant at *p* < 0.05. Transcriptomic and metabolomic visualizations, including Venn diagrams, heat maps, and volcano plots, were generated using the Omicsmart online platform (www.omicsmart.com, accessed on 25 January 2025). Figures were prepared using OriginPro 2025 and Adobe Illustrator 2025.

## 5. Conclusions

This study investigated the physiological responses, Cu uptake and translocation, antioxidant defense, and transcriptomic and metabolomic responses of *Pistia stratiotes* to CuO NP exposure. At the lowest exposure concentration (T_1_), photosynthetic performance was largely maintained, accompanied by increased photosynthetic pigment contents and changes in antioxidant enzyme activities. Root Cu concentrations increased across all CuO NP treatments, whereas the translocation factor (TF) decreased, indicating reduced Cu translocation from roots to shoots. At higher concentrations (T_3_ and T_4_), photosynthetic performance declined and superoxide anion (O_2_·^−^) accumulation increased. Changes in antioxidant enzyme activities, together with increased leaf GSH and Cys contents, further highlighted the involvement of antioxidant defense responses under CuO NP exposure.

Multi-omics analyses further identified cell wall-related carbohydrate metabolism and carotenoid-associated metabolism as prominent molecular responses to CuO NP exposure. Differential expression of pectin-related genes in the pentose and glucuronate interconversions pathway, together with changes in carotenoid metabolite abundances and the expression of genes associated with ABA biosynthesis from carotenoid precursors, points to cell wall remodeling and carotenoid-related metabolic and hormonal responses as potential components of the plant’s response to CuO NPs. These findings provide molecular context for the observed physiological changes, although the specific contributions of these pathways to Cu retention and photosynthetic responses require further investigation. Furthermore, because dissolved Cu was not quantified and no soluble-Cu control was included, the observed responses cannot be attributed exclusively to intact nanoparticles. Overall, this study links concentration-dependent physiological responses with changes in cell wall-related and carotenoid-associated pathways, providing a basis for further evaluating the phytoremediation potential of *P. stratiotes* in aquatic environments contaminated with copper and CuO NPs.

## Figures and Tables

**Table 1:** Cu uptake, translocation, and accumulation in *Pistia stratiotes* after exposure to different concentrations of CuO NPs.

Treatment	Cu Concentration (mg·kg^–1^ DW)		BCF (L·kg^−1^ DW)	TF
	Leaf	Root		
CK	2.08 ± 0.11 c	3.23 ± 0.15 e	152.69 ± 12.56 b	0.65 ± 0.06 a
T_1_	17.74 ± 1.66 c	346.16 ± 26.56 d	613.70 ± 62.17 a	0.15 ± 0.01 b
T_2_	45.77 ± 15.17 b	747.47 ± 61.33 c	99.83 ± 12.55 b	0.10 ± 0.02 c
T_3_	80.38 ± 6.31 a	1025.38 ± 8.74 b	58.56 ± 1.89 c	0.11 ± 0.01 c
T_4_	87.22 ± 9.45 a	1342.92 ± 100.07 a	40.18 ± 3.23 c	0.09 ± 0.01 c

Values are presented means ± SE (*n* = 3). Different lowercase letters within a column indicate significant differences among treatments based on Duncan’s multiple range test (*p* < 0.05).

**Table 2:** Criteria for assessing CuO NP stress injury in *Pistia stratiotes*.

Injury Score	Observed Morphological Characteristics
0	No visible toxic symptoms.
1	Newly grown leaves appear normal; some older leaves display slight wilting, yellowing, and curling at the edges.
2	Some newly grown leaves exhibit edge curling; older leaves show more pronounced wilting, yellowing, and edge curling.
3	More than half of the leaves exhibit wilting and rotting.
4	More than four-fifths of the leaves exhibit wilting and rotting.
5	Complete plant death.

## Data Availability

The data supporting the findings of this study are available in the article, its Supplementary Materials, and the NCBI Sequence Read Archive (SRA). The raw RNA-seq data are publicly available under BioProject accession number PRJNA1544582 (SRA Study accession number SRP746537; run accession numbers SRR41225141–SRR41225149). Further inquiries regarding the data can be directed to the corresponding author.

## Supplementary Materials

The following supporting information can be downloaded at: https://www.mdpi.com/article/10.3390/plants15213271/s1, Figure S1: Top 10 DAMs in the CK-vs.-T_1_, CK-vs.-T_3_, and T_1_-vs.-T_3_ comparison groups; Figure S2: Expression of DEGs involved in photosynthesis-antenna proteins in CK-vs.-T3 comparison; Figure S3: Iron and sulfur contents in leaves and calcium and zinc contents in roots after 14 d of CuO NP exposure. Figure S4: Differentially expressed TFs; Table S1. Top 10 DAMs in the CK-vs.-T_1_, CK-vs.-T_3_, and T_1_-vs.-T_3_ comparison groups; Table S2: Statistics of base information after transcriptome data filtering; Table S3: Comparison of reference genome statistics; Table S4: Integrated analysis of transcriptomics and metabolomics; Table S5: Mercator-annotated DEGs for MapMan pathway mapping; Table S6: The primer sequences for qRT-PCR.

## Appendix A. Determination of Antioxidant System and Ascorbate–Glutathione Cycle Parameters

Leaf samples collected at 0, 7, and 14 days after treatment were used to determine the MDA, reactive oxygen species (ROS), antioxidant enzyme activities, ascorbate (Asc), glutathione (GSH), and cysteine (Cys) contents.

### Appendix A.1. Determination of Malondialdehyde and Soluble Sugar Contents

The malondialdehyde (MDA) and soluble sugar contents were determined using a modified thiobarbituric acid (TBA) method according to Janero [107]. Briefly, 0.3 g of fresh leaf tissue was homogenized in 1 mL of 5% (*w*:*v*) trichloroacetic acid (TCA), brought to a final volume of 5 mL with 5% TCA, and centrifuged at 4500× *g* for 30 min at 4 °C. Subsequently, 2 mL of the extract was mixed with 2 mL of 0.67% (*w*:*v*) TBA solution and incubated at 100 °C for 30 min. After cooling and centrifugation, the absorbance was measured at 450, 532, and 600 nm using an Infinite 200 PRO microplate reader (Tecan, Männedorf, Switzerland).

### Appendix A.2. Determination of Reactive Oxygen Species Contents

The superoxide anion (O_2_·^−^) content was determined using a modified hydroxylamine oxidation method according to Elstner and Heupel [108]. Briefly, 0.3 g of fresh leaf tissue was homogenized in 5 mL of precooled 50 mmol·L^−1^ phosphate buffer (pH 7.8) containing 1% (*w*:*v*) polyvinylpyrrolidone. The 6.0-mL reaction system contained 0.5 mL of extract, 0.5 mL of phosphate buffer, 1.0 mL of 1 mmol·L^−1^ hydroxylamine hydrochloride, 2.0 mL of 17 mmol·L^−1^ sulfanilic acid, and 2.0 mL of 7 mmol·L^−1^ α-naphthylamine. After incubation at 25 °C for 1 h and then for an additional 20 min, the absorbance was measured at 530 nm.

The hydrogen peroxide (H_2_O_2_) content was determined using a modified titanium sulfate colorimetric method according to Zhou et al. [109]. Leaf tissue was extracted with 5 mL of precooled acetone containing 5% (*w*:*v*) trichloroacetic acid (TCA). The reaction system contained 250 μL of extract, 25 μL of 20% (*w*:*v*) titanium sulfate prepared in concentrated hydrochloric acid, and 50 μL of concentrated ammonia. After centrifugation at 4000× *g* for 10 min at 25 °C, the precipitate was dissolved in 250 μL of 2 mol·L^−1^ sulfuric acid, and the absorbance was measured at 415 nm. H_2_O_2_ standards were prepared using acetone containing 5% (*w*:*v*) TCA.

### Appendix A.3. Determination of Antioxidant Enzyme Activities

For enzyme assays, 0.3 g of fresh leaf tissue was homogenized in 5 mL of precooled 50 mmol·L^−1^ phosphate buffer (pH 7.8) containing 1% (*w*:*v*) polyvinylpyrrolidone and centrifuged to obtain the crude enzyme extract.

Peroxidase (POD) activity was determined using a modified guaiacol method according to Rosalki [110]. The 2.9-mL reaction mixture contained 1.0 mL of 50 mmol·L^−1^ phosphate buffer (pH 7.0), 0.9 mL of 0.2% (*w*:*v*) guaiacol, and 1.0 mL of 0.3% (*v*:*v*) H_2_O_2_ solution. After the addition of 0.1 mL of crude enzyme extract, the increase in absorbance at 470 nm was recorded from 0.5 to 3.5 min.

Catalase (CAT) activity was determined using a modified ultraviolet absorption method according to Lu et al. [111]. The reaction mixture contained 1.5 mL of 50 mmol·L^−1^ phosphate buffer (pH 7.8), 1.0 mL of distilled water, 0.3 mL of 0.3% (*v*:*v*) H_2_O_2_ solution, and 0.2 mL of crude enzyme extract. The decrease in absorbance at 240 nm was recorded from 0.5 to 3.5 min.

Ascorbate peroxidase (APX) activity was determined using the ascorbate oxidation method according to Nakano and Asada [112]. The 2.9-mL reaction mixture contained 50 mmol·L^−1^ phosphate buffer (pH 7.8), 0.1 mmol·L^−1^ EDTA, 0.3 mmol·L^−1^ ascorbate, and 0.06 mmol·L^−1^ H_2_O_2_ solution. After the addition of 0.1 mL of crude enzyme extract, the decrease in absorbance at 290 nm was recorded from 10 to 70 s.

Dehydroascorbate reductase (DHAR) activity was determined using the DHA reduction method according to Dalton et al. [113]. The 2.9-mL reaction mixture contained 50 mmol·L^−1^ phosphate buffer (pH 7.0), 0.1 mmol·L^−1^ EDTA, 2.5 mmol·L^−1^ GSH, and 0.1 mmol·L^−1^ DHA. After the addition of 0.1 mL of crude enzyme extract, the decrease in absorbance at 265 nm was recorded from 10 to 70 s.

Glutathione reductase (GR) activity was determined using a modified NADPH oxidation method according to Carlberg and Mannervik [114]. The 2.0-mL reaction mixture contained 1.0 mL of oxidized glutathione solution, 0.7 mL of 50 mmol·L^−1^ phosphate buffer (pH 7.8), 0.2 mL of crude enzyme extract, and 0.1 mL of 2 mmol·L^−1^ NADPH solution. The decrease in absorbance at 340 nm was recorded from 0.5 to 3.5 min.

### Appendix A.4. Determination of Ascorbate and Glutathione Contents

Leaf tissue (0.3 g) was homogenized in precooled 5% (*w*:*v*) sulfosalicylic acid and centrifuged to obtain the extracts.

The total ascorbate (total Asc) content was determined according to Kampfenkel et al. [115], with modifications. The 1761-μL reaction system contained 150 μL of extract, 36 μL of 1.84 mol·L^−1^ triethanolamine, 375 μL of 50 mmol·L^−1^ phosphate buffer (pH 7.5) containing 2.5 mmol·L^−1^ ethylenediaminetetraacetic acid, 75 μL of 10 mmol·L^−1^ dithiothreitol, 75 μL of 0.5% (*w*:*v*) N-ethylmaleimide, 300 μL of 10% (*w*:*v*) trichloroacetic acid, 300 μL of 44% (*v*:*v*) phosphoric acid, 300 μL of 4% (*w*:*v*) 2,2′-bipyridyl in ethanol, and 150 μL of 3% (*w*:*v*) FeCl_3_ solution. After incubation at 40 °C for 40 min, the absorbance was measured at 525 nm. An Asc standard curve was prepared using Asc standards at concentrations ranging from 0 to 1.00 mmol·L^−1^.

The total glutathione (total GSH) content was determined using a modified dithiobis(2-nitrobenzoic acid) (DTNB) colorimetric method according to [116]. The 2.5-mL reaction mixture contained 1.0 mL of extract, 1.0 mL of 0.1 mol·L^−1^ phosphate buffer (pH 7.7), and 0.5 mL of 4 mmol·L^−1^ DTNB solution. After incubation at 25 °C for 10 min, the absorbance was measured at 412 nm. The total GSH content was calculated from the corresponding standard curve.

### Appendix A.5. Determination of Cysteine Content

The cysteine (Cys) content was determined using a commercial visible spectrophotometric assay kit (CYS-F48-N(1620), Nanjing Kemerson Biotechnology Co., Ltd., Nanjing, China) according to the manufacturer’s instructions. Briefly, approximately 0.1 g of leaf tissue was homogenized with 1 mL of extraction reagent 1 under ice-cold conditions and centrifuged at 8000× *g* for 10 min at 4 °C. Then, 100 μL of supernatant was mixed with 500 μL of reagent 2 and 500 μL of reagent 3 and incubated at room temperature for 15 min. The absorbance was measured at 600 nm, and the Cys content was calculated from the standard curve.
