# Supplementary material for: Integrated Physiological, Transcriptomic, and Metabolomic Analyses Reveal Coordinated Responses of the Invasive Plant Pistia stratiotes to Copper Oxide Nanoparticles
Source: Plants (Basel). 2026 Oct 23;15(21):3271. doi: 10.3390/plants15213271 (PMC13765993; doi:10.3390/plants15213271)
Supplement: Supplementary file 1 [file plants-15-03271-s001.zip › Supplementary Figures.pdf]

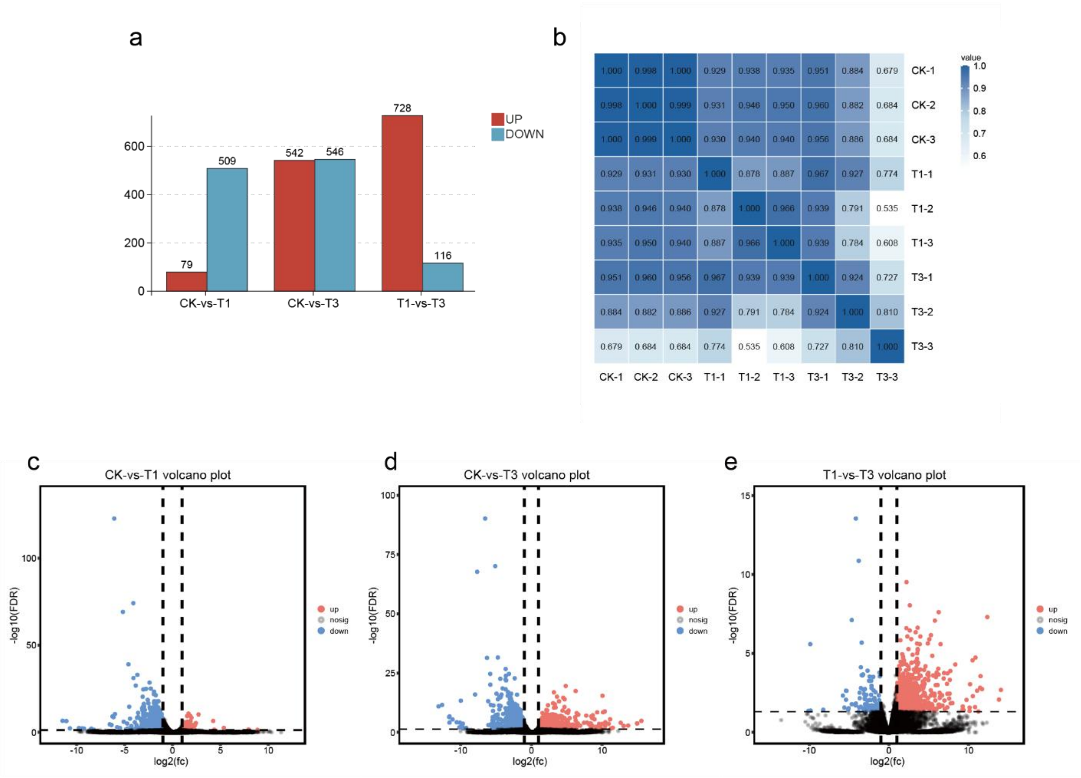

Figure S1. Changes in DEGs expression. (a) Up-regulation and down-regulation of DEGs, red represents up-regulation, blue represents down-regulation. (b) Heat map of sample correlation. (c, d, e) Volcano plots of DEGs up-regulation and down-regulation.

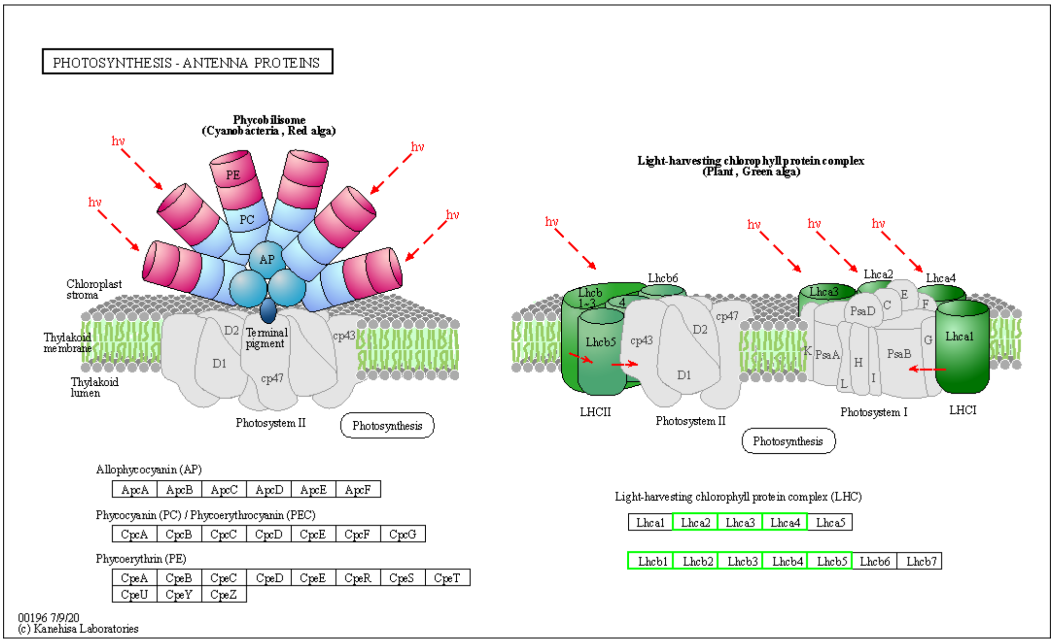

Figure S2. Expression of DEGs involved in photosynthesis-antenna proteins in CK vs T_3 comparison. Genes with green borders are differentially down-regulated genes annotated to this pathway, whereas genes with red borders are differentially up-regulated genes annotated to this pathway.

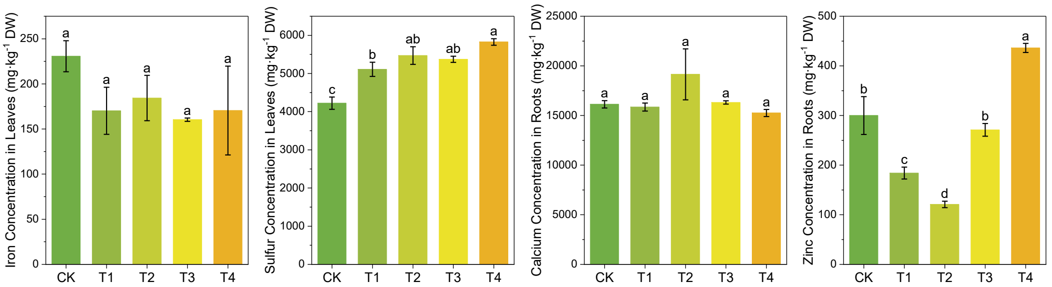

Figure S3. Iron and sulfur contents in leaves and calcium and zinc contents in roots after 14 d of CuO NPs exposure. Different lowercase letters indicate significant differences among treatments ($P < 0.05$).

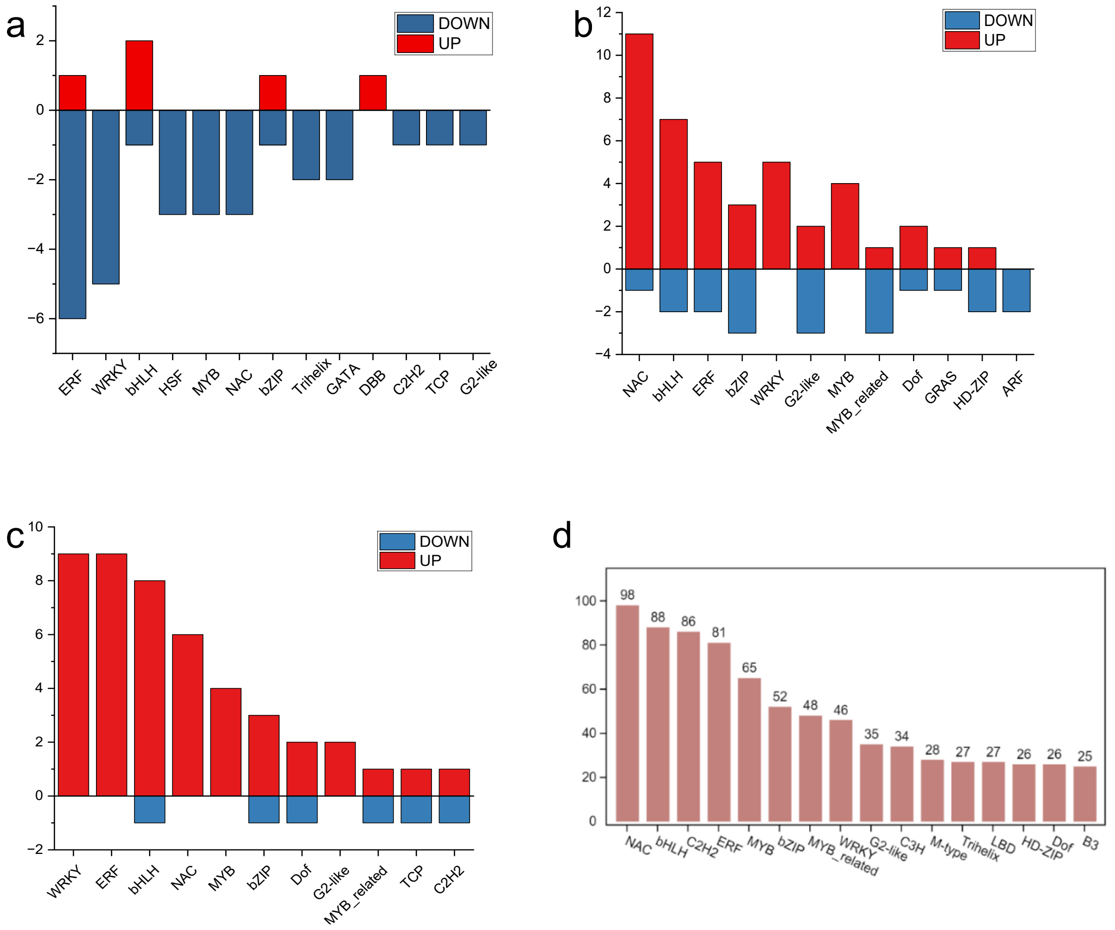

Figure S4. Differentially expressed TFs in (**a**) CK vs T_1, (**b**) CK vs T_3, (**c**) T_1 vs T_3 groups and (**d**) the top 15 distribution of the TFs.
